# Aminopolyols from Carbohydrates: Amination of Sugars and Sugar‐Derived Tetrahydrofurans with Transaminases

**DOI:** 10.1002/anie.201813712

**Published:** 2019-02-14

**Authors:** Fabiana Subrizi, Laure Benhamou, John M. Ward, Tom D. Sheppard, Helen C. Hailes

**Affiliations:** ^1^ Department of Chemistry University College London 20 Gordon Street London WC1H 0AJ UK; ^2^ Department of Biochemical Engineering University College London Bernard Katz Building London WC1E 6BT UK

**Keywords:** aminopolyols, biocatalysis, biomass, carbohydrates, enzymes

## Abstract

Carbohydrates are the major component of biomass and have unique potential as a sustainable source of building blocks for chemicals, materials, and biofuels because of their low cost, ready availability, and stereochemical diversity. With a view to upgrading carbohydrates to access valuable nitrogen‐containing sugar‐like compounds such as aminopolyols, biocatalytic aminations using transaminase enzymes (TAms) have been investigated as a sustainable alternative to traditional synthetic strategies. Demonstrated here is the reaction of TAms with sugar‐derived tetrahydrofuran (THF) aldehydes, obtained from the regioselective dehydration of biomass‐derived sugars, to provide access to cyclic aminodiols in high yields. In a preliminary study we have also established the direct transamination of sugars to give acyclic aminopolyols. Notably, the reaction of the ketose d‐fructose proceeds with complete stereoselectivity to yield valuable aminosugars in high purity.

Aminated carbohydrates such as aminosugars, iminocyclitols, and other linear or cyclic polyhydroxylated amines are of particular interest as carbohydrate mimetics for the treatment of diabetes and viral infections, as antitumor or immunosuppressive agents,^1^, ^2^, ^3^ and as monomers in biopolymer formation.^1^, ^2^, ^4^ Chiral aminopolyols therefore represent an interesting class of higher‐value products that could be accessed from renewable carbohydrate feedstocks such as sugar beet pulp.^5^, ^6^, ^7^ The amination of highly functionalized carbohydrates by traditional synthetic strategies typically requires regioselective control using complex chemical strategies^3^ and protection‐deprotection steps which decrease the overall atom economy.^1^, ^2^, ^8^ Furthermore, using traditional reductive amination methods, alkylation events typically occur, resulting in the formation of undesired secondary or tertiary amine products.^9^, ^10^ As an alternative approach, biocatalytic methods such as transaminases (TAms),^11^, ^12^ imine reductases,^13^, ^14^, ^15^, ^16^ or amine dehydrogenases could be used.^17^ Biocatalytic aminations can be significantly more sustainable than chemical approaches, but to date, the biocatalytic amination of sugars to provide access to aminopolyols has not been reported.

TAms were selected as suitable enzymes as they have been reported to accept a wide range of substrates including cyclic ketones,^18^ aromatic ketones,^19^, ^20^, ^21^ steroids,^22^ heterocyclic compounds,^6^, ^23^ hydroxyketones,^24^ and dihydroxyketones,^25^, ^26^ and are used industrially, for example, in the synthesis of Sitagliptin.^27^ Many transaminases have been developed for the synthesis of lipophilic amines, but very few have been described to accept either polyhydroxylated ketones^25^ or aldehydes, and to the best of our knowledge, none have been reported in the literature to accept reducing sugars. A further challenge is that sugars are present almost entirely in the hemiacetal form in solution, with very little free carbonyl present to undergo reaction with the enzyme (Scheme 1). As a consequence, in this work we initially explored the reaction of TAm enzymes with sugar‐derived tetrahydrofuran (THF) aldehydes, generated by the regioselective dehydration of biomass‐derived sugars,^28^ to provide access to cyclic aminopolyols (Scheme 1 A). These compounds were found to be excellent substrates for a variety of TAm enzymes, giving the corresponding amines in excellent yields. Subsequently, in a preliminary study we also demonstrated the direct transamination of several sugars to give acyclic aminopolyols, which were obtained with excellent diastereoselectivities from a ketosugar (Scheme 1 B).

**Scheme 1:**
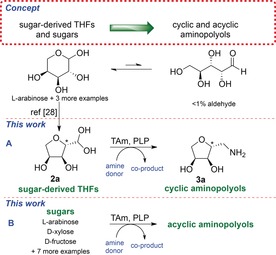
Previous approach to sugar‐derived THFs from sugars (e.g., **2 a**). This work using: a) TAms to generate amine‐THFs (e.g. **3 a)**. b) TAms to generate acyclic aminopolyols.

The preparation of the amine **3 a**, by catalytic hydrogenation of the corresponding dimethylhydrazone, gave the Boc‐derivative **4 a** in a moderate 60 % yield.^28^ While this is a useful method to access the aminodiol, significant problems were encountered on scaling up the chemistry. We therefore elected to study a more sustainable approach by exploring the reaction of TAm enzymes with the cyclic aldehydes **2 a**–**d**, prepared from l‐arabinose, d‐ribose, d‐xylose, and l‐rhamnose, respectively (Scheme 2).^28^ This three‐step synthesis was efficient with overall yields ranging from 77–91 %, with the diastereomeric ratio (due to C2 isomers) varying considerably between the different sugar derivatives.

**Scheme 2:**
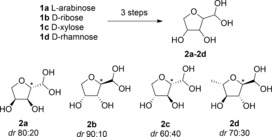
Aldehydes prepared: **2 a**, 77 % from l‐arabinose, **2 b**, 79 % from d‐ribose, **2 c**, 91 % from d‐xylose, **2 d**, 81 % from l‐rhamnose. Major isomer shown at C2 (starred*).

Initially, 11 TAms were selected from our library at UCL, based upon high activities previously observed in other screening programs. The preliminary experiments were carried out using crude cell lysates and a colorimetric assay with the amine donor 2‐(4‐nitrophenyl)ethan‐1‐amine (**5**), which produces a red precipitate in successful amination reactions (Figure 1 A).^29^ The colorimetric assay, initially conducted on **2 a**, showed good activity for eight of the TAms selected from our library (see Figure S4 in the Supporting Information). From this initial screen three were selected for further study, two *S*‐selective TAms, *Chromobacterium violaceum* TAm (*Cv*‐TAm)^30^ and *Rhodobacter sphaeroides* TAm (*Rh*‐TAm),^25^ as they have been shown to be versatile enzymes for many substrates, and the *R*‐selective *Mycobacterium vanbaalenii* TAm (*Mv*‐TAm),^31^ because of its complementary stereoselectivity. These three TAms were then screened against **2 a**–**d** and a strong red color was observed with all enzymes, indicating good levels of substrate acceptance (Figure 1 B).

**Figure 1:**
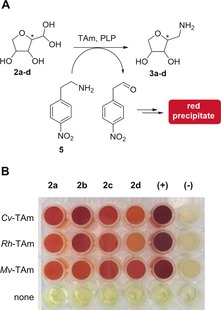
Colorimetric assay on compounds **2 a**–**d** (10 mm) using **5** as the amine donor (25 mm). Pyruvate was used as a positive control (+) and buffer solution as a negative control (−).

Activities of the three TAms towards **2 a**–**d** were also confirmed using either (*R*)‐ or (*S*)‐α‐methylbenzylamine (MBA) as the amine donor. The best results were obtained with *Cv*‐TAm and *Rh*‐TAm on **2 a** and **2 b**, which gave conversion yields of 60–63 % using (*S*)‐MBA while up to 27 % yield was achieved with *Mv*‐TAm under the same reaction conditions with (*R*)‐MBA (Figure 2). Moreover, the reaction seemed to be complete within 3 hours for *Cv*‐TAm and *Rh*‐TAm with **2 a** and **2 b**, while reactions with substrates **2 c** and **2 d** showed slower kinetics (see Figure S5) and lower yields ranging from 40–50 %. Notably, conversion yields with *Mv*‐TAm were similar for all compounds (**2 a**–**d**). With the aim of enhancing yields further, an alternative amine donor, isopropylamine (IPA; **6**), was used (Table 1). With both *Rh*‐TAm and *Cv*‐TAm the yields improved significantly (up to 91 % for **2 b**). These reaction conditions also gave improved yields with *Mv*‐TAm, with all the substrates giving yields of between 60 and 81 %. For both **2 a** and **2 b** all the selected TAms appeared to preferentially accept the major *anti* isomer while the *syn* isomer was converted more slowly into the corresponding amine leading to product yields of up to 91 % and diastereomeric ratios of up to 95:5. In the same way *Rh*‐TAm more readily accepted the *anti* isomer of **2 c** (d.r. 60:40), producing **3 c** with an enhanced d.r. (70:30). Interestingly *Cv*‐TAm more readily accepted the minor *syn* isomer of **2 c**, which was almost all converted in the first 30 minutes as shown in time‐course experiments using either HPLC or NMR spectroscopy to monitor the reaction (see Figures S6 and S7). However, *anti*‐**2 c** is also converted into the corresponding amine product *anti*‐**3 c**, which is the major product at the end of the reaction. By comparison, *Mv*‐TAm had a clear preference for converting the minor isomer *syn*‐**2 c** (20:80 d.r. after 4 h), although after 24 hours the ratio was 35:65 (see Figure S6). In contrast, in the case of the rhamnose‐derived THF **2 d** the two isomers were converted at similar rates, with all three enzymes, to give the amines **3 d** in a similar isomeric ratio to the starting material in 75–84 % yield. The THF‐amines **3 a**–**d** are valuable biomass‐derived synthons and biocatalytic syntheses were investigated on an enzyme preparative scale (50 mm) using either *Cv*‐TAm or *Rh*‐TAm. Under these reaction conditions, **2 a**–**c** were converted quantitatively and only traces of the starting material were detected in the case of **2 d**. The aminated products were isolated by derivatization with di‐*tert*‐butyl dicarbonate (Boc_2_O), to give the corresponding derivatives **4 a**–**d** in 58–76 % yield (Table 1). After deprotection, **3 a**–**d** were quantitatively recovered as the hydrochloride salt in high purity.

**Figure 2:**
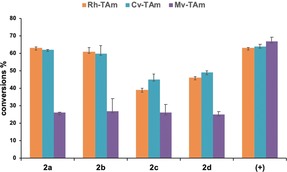
Assays using 20 mm (*S*)‐MBA (*Cv*‐TAm and *Rh*‐TAm) or (*R*)‐MBA (*Mv*‐TAm) as amine donor with the three selected TAms towards **2 a**–**d** (5 mm). Pyruvate was used as a positive control (+) and buffer solution as a negative control (−).

**Table 1:** Yields for the TAm reactions using **6** (10 equiv) and **2 a**–**d** (25 mm) to give **3 a**–**d**. 
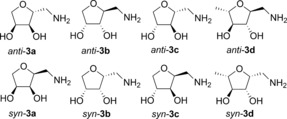

Starting material	**2 a** (80:20)	**2 b** (90:10)	**2 c** (60:40)	**2 d** (70:30)
*Cv*‐TAm	*anti*‐**3 a** 84 % (90:10)	*anti*‐**3 b** 91 % (95:5) 76 %^[a]^	*anti*‐**3 c** 74 % (65:35)	*anti*‐**3 d** 81 % (75:25) 58 %^[a]^
				
*Rh*‐TAm	*anti*‐**3 a** 75 % (90:10) 71 %^[a]^	*anti*‐**3 b** 85 % (95:5)	*anti*‐**3 c** 67 % (70:30) 69 %^[a]^	*anti*‐**3 d** 84 % (65:35)
				
*Mv*‐TAm	*anti*‐**3 a** 56 % (85:15)	*anti*‐**3 b** 47 % (95:5)^[b]^ 70 % (95:5)	*syn*‐**3 c** 31 % (20:80)^[b]^ 60 % (35:65)	*anti*‐**3 d** 75 % (70:30)

The reactions were run at 37 °C for 24 h unless indicated otherwise. The major product is indicated with the yield and diastereomeric ratio (d.r. *anti/syn*) calculated by HPLC analysis after derivatization with FmocCl. [a] Yield of the isolated Boc‐protected amine (**4a**–**d**) obtained on a preparative scale (50 mm). [b] Reaction time 4 h.

Having successfully demonstrated the amination of sugar‐derived THFs to access cyclic aminopolyols, the amination of reducing sugars using TAms was also explored. In preliminary screens, class‐VI and class‐III TAms from our UCL library were selected. Class‐VI TAms, known as sugar aminotransferases, largely use the amine donor l‐glutamate and accept activated keto‐hexoses, while class‐III TAms such as *Cv*‐TAm are characterized by their broad substrate acceptance.^32^ More than 30 TAms (including three from a metagenomic study)^33^ were screened as crude cell lysates against l‐arabinose using **5** as the amine donor. No hits were identified with the class‐VI TAms, perhaps reflecting that either another amine donor was required or that they typically convert a cyclic non‐anomeric ketone. However, several TAms, notably *Rh*‐TAm and the enzyme encoded by pQR2191, accepted l‐arabinose using **5** as the amine donor (Figure 3). Other sugars were also tested, and d‐ribose was generally better accepted than l‐arabinose, whilst d‐xylose and l‐rhamnose were accepted only by *Rh*‐TAm and the enzyme encoded by pQR2191, respectively. Notably, d‐fructose, a ketosugar, was accepted by *Mv*‐TAm and the TAm encoded by pQR2191 using **5** as the amine donor.

**Figure 3:**
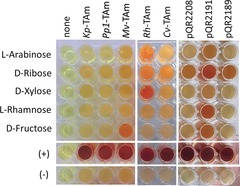
Assays of TAm enzymes using **5** (25 mm) with a selection of sugar substrates (5 mm) at 30 °C, pyruvate (+) was used as a positive control and buffer (−) as a negative control. *Pseudomonas putida* TAm (*Pp1*‐TAm pQR427) and *Klebsiella pneumoniae* TAm (*Kp*‐TAm)^25^ were representative of many of the TAms which showed no activities towards the sugar substrates. Enzymes encoded by pQR2189, pQR2191, and pQR2208 have been reported previously.^33^

The variation in sugar conversions could reflect the fact that certain stereochemistries are not accepted in the TAm active site. However, they will also be significantly influenced by the equilibria between acyclic pyranose and furanose forms in water. Sugar mutarotation is also influenced by temperature and pH.^34^ On this basis we explored the effect of these parameters on the conversion of selected sugars using *Cv*‐TAm and *Rh*‐TAm. The results suggested improved activity at more basic pH values and at higher temperatures. This finding was consistent with the observation that the proportion of the acyclic form of the sugar typically increases with temperature.^34^ It has also been reported that when in DMSO the pyranose–furanose equilibria change and for some sugars such as arabinose and fructose, a higher proportion of the furanose form exists.^35^ Indeed, the yield doubled to 15 % with d‐ribose [(*S*)‐MBA and *Cv*‐TAm] when the assay was conducted in the presence of 5 % of DMSO at 30 °C and pH 8. This result suggested that robust thermostable TAms able to tolerate high proportions of DMSO may give enhanced reactivities. In particular, three of the selected TAms (namely those encoded by pQR2189, pQR2191, and pQR2208) have been reported to have excellent tolerance towards DMSO and higher temperatures,^33^ and these enzymes were therefore used in up to 40 % v/v DMSO. Satisfyingly, two of the three TAms selected, encoded by pQR2189 and pQR2191, showed good activity towards almost all the aldose sugars tested with DMSO as cosolvent at 45 °C (see Figures S8 and S9). With these improved reaction conditions we extended the screen to other sugars including d‐galacturonic acid (d‐GalAc) and ketose sugars with different chain lengths (e.g. d‐ribulose, l‐sorbose and d‐tagatose, and l‐glucoheptulose; Figure 4; see Figure S10). The latter was efficiently prepared by a one‐step reaction catalyzed by a mutant transketolase enzyme starting from l‐arabinose.^36^, ^37^

**Figure 4:**
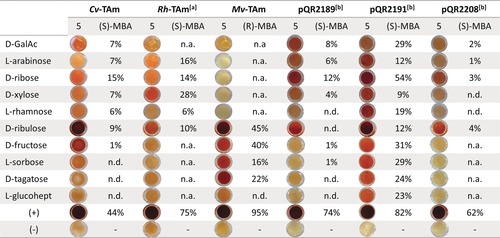
TAm catalyzed reactions of sugars using **5**, and either (*S*)‐ or (*R*)‐MBA as amine donors. Reaction conditions: amine donor **5** (25 mm) or MBA (20 mm), sugar (10 mm or 5 mm), KPi buffer pH 8 (50 mm) and clarified cell extract, 45 °C, 400 rpm, 24 h. Buffer was used as a negative control (−) and pyruvate as a positive control (+). [a] Reaction at 30 °C. [b] Reaction in the presence of 25 % of DMSO.

Quantification of the TAm reactions was carried out using MBA and acetophenone detection by HPLC. This method indicated conversion yields of up 54 % for d‐ribose, 29 % for d‐GalAc and l‐sorbose, 28 % for d‐xylose, and about 23 % for d‐tagatose and l‐glucoheptulose. Notably, *Mv*‐TAm showed a conversion yield of 40 % with d‐fructose at 45 °C when (*R*)‐MBA was used as the amine donor. Generally, the ketosugars were well accepted by *Mv*‐TAm and the TAm encoded by pQR2191, although l‐glucoheptulose was only accepted by pQR2191. Use of the cosolvent DMSO also seemed to be less relevant when a keto sugar was used as the substrate as high conversions could still be obtained in its absence. The acceptance of d‐GalAc (Figure 4) was interesting as this produces an ω‐aminoacid with potential as a monomer for the preparation of polyhydroxy polyamides as analogues of Nylon‐6.^38^ Upon cyclization, it would also provide an advanced precursor for the preparation of a potent polyhydroxyazepane glycosidase inhibitor.^39^

Linear aminopolyols are valuable products and direct syntheses from sugars using TAm were therefore investigated on a biocatalytic preparative scale and isolated using a Dowex 50WX8 ion‐exchange resin.^8^ *Rh*‐TAm in reactions with l‐arabinose and d‐xylose (at 25 mm) and IPA (see the Supporting Information), gave **7** 8 in 42 % yield and **8** in 79 % yield (Scheme 3). The reaction of d‐fructose with *Mv*‐TAm was scaled up, using either (*R*)‐MBA or IPA as the amine donor, to a 25 mm scale at 45 °C. ^1^H NMR analysis indicated a 50 % yield of the corresponding aminopolyol as a single diastereoisomer. The product **9** was isolated in 40 % yield from the reaction with IPA. The configuration of the new stereogenic center was assigned as *R* in agreement with reported data,^40^ which is consistent with the known selectivity of the *Mv*‐TAm. With the aim of isolating the C2 epimer we also scaled up the reaction with d‐fructose and the enantiocomplementary TAm encoded by pQR2191 and IPA. Again, the reaction was highly stereoselective and e*pi*‐**9** was isolated in 21 % yield.^41^ These reactions constitute the first report of the direct amination of reducing sugars using TAm enzymes to give access to potentially valuable aminopolyols on a preparative scale.

**Scheme 3:**
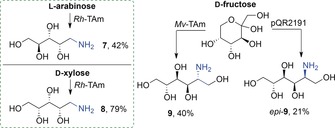
Synthesis of aminopolyols from l‐arabinose, d‐xylose, and d‐fructose using TAms and either **6** or MBA as amine donors.

In summary, the transamination of sugar‐derived THFs and sugars available from biomass has been achieved in high yield, providing access to valuable cyclic and acyclic aminopolyols. Several TAms were identified to readily accept the sugar‐derived THF‐aldehydes, and using IPA as an amine donor, yields of 58–76 % were obtained. Moreover, the direct transamination of reducing sugars has been achieved, with transamination conversions for some sugar substrates increasing at higher temperatures and with the addition of DMSO. The first preparative scale transaminase reactions of reducing sugars have also been achieved, with the amination of d‐xylose and l‐arabinose. d‐fructose was also converted stereoselectively into either **9** or *epi*‐**9** using enantiocomplementary TAms. The aminopolyol **9** is an ingredient in cosmetic formulations, and an advanced precursor for the preparation of the potent α‐glycosidase inhibitor 1‐deoxynojirimycin.^42^

## Conflict of interest

The authors declare no conflict of interest.

## Supporting information

As a service to our authors and readers, this journal provides supporting information supplied by the authors. Such materials are peer reviewed and may be re‐organized for online delivery, but are not copy‐edited or typeset. Technical support issues arising from supporting information (other than missing files) should be addressed to the authors.

Supplementary
