# Supplementary material for: Aminopolyols from Carbohydrates: Amination of Sugars and Sugar‐Derived Tetrahydrofurans with Transaminases
Source: Angew Chem Int Ed Engl. 2019 Feb 14;58(12):3854–8. doi: 10.1002/anie.201813712 (PMC6492202; doi:10.1002/anie.201813712)
Supplement: Supplementary file 1 — Supplementary [file ANIE-58-3854-s001.pdf]

Angewandte Chemie
Eine Zeitschrift der Gesellschaft Deutscher Chemiker

Supporting Information

# Aminopolyols from Carbohydrates: Amination of Sugars and Sugar-Derived Tetrahydrofurans with Transaminases

*Fabiana Subrizi, Laure Benhamou, John M. Ward, Tom D. Sheppard, and Helen C. Hailes**

anie_201813712_sm_miscellaneous_information.pdf

## Author Contributions

H.H. Conceptualization: Lead; Investigation: Lead; Methodology: Supporting; Project administration: Lead; Supervision: Lead; Writing—original draft: Lead; Writing—review & editing: Lead
F.S. Conceptualization: Supporting; Investigation: Lead; Methodology: Lead; Writing—original draft: Lead
L.B. Investigation: Supporting; Methodology: Supporting; Writing—review & editing: Supporting
J.W. Conceptualization: Supporting; Investigation: Supporting; Methodology: Supporting; Supervision: Supporting; Writing—review & editing: Supporting
T.S. Investigation: Supporting; Methodology: Supporting; Supervision: Supporting; Writing—review & editing: Lead.

**Table of contents:**


| | | |
|---|---|---|
| I. | General experimental and analytic methods | S2 |
| II. | Analytical HPLC chromatograms | S5 |
| III. | Supplementary Data and Figures | S8 |
| IV. | Chemical syntheses and preparative scale biocatalytic reactions | S15 |
| V. | NMR spectra | S30 |
| VI. | References | S47 |

## I. General Experimental and analytic methods

The solvents and chemicals were purchased from Sigma-Aldrich or Fluorochem and were used as supplied. Column chromatography was carried out using BDH (40-63 εm) silica gel and analytical thin layer chromatography was carried out using Merck Kieselgel aluminium-backed plates coated with silica gel. Compounds were visualised using combinations of UV (254 nm) or potassium permanganate. $^{1}$H and $^{13}$C NMR spectra were recorded respectively at 400 MHz and 100 MHz on a Bruker Avance 400 spectrometer, 500 MHz and 125 MHz on a Bruker Avance 500 or at 600 MHz and 150 MHz on a Bruker Avance 600 spectrometer in the stated solvent. Chemical shifts (in ppm) are quoted relative to tetramethylsilane and referenced to residual protonated solvent. Coupling constants (*J*) are measured in Hertz (Hz) and multiplicities for $^{1}$H NMR coupling are shown as s (singlet), d (doublet), t (triplet), and m (multiplet). HPLC analyses were performed on an Agilent 1260 Infinity provided with a 1260 VWD detector. Infrared (IR) spectra were recorded on a Perkin-Elmer spectrum 100 FT-IR spectrometer as thin films. Mass spectra were obtained using either a VG70-SE or MAT 900XP spectrometer at the Department of Chemistry, University College London. All optical rotations were measured on a Perkin-Elmer 343 polarimeter with a path length of 1 dm.

**Transaminase expression and preparation.** Overnight cultures (10 mL) of the TAms in *E. coli* from the UCL TAm library were prepared in 2xTY broth (16 g/L tryptone, 10 g/L yeast extract, 5 g/L NaCl) supplemented with kanamycin (50 εg/mL) and incubated overnight at 37 °C. Cells were subcultured using 1% v/v inoculum in 2 L shake flasks containing 500 mL of the same supplemented broth at 37 °C and 250 rpm. Transaminases were induced with 1 mM of IPTG when growing in the early exponential phase ($OD_{600}$ = 0.5 - 0.7), and the temperature was dropped to 30 °C until harvesting. Cells were harvested by centrifugation after 5 h of induction, and stored at ī 20 °C. When needed, the cell pellet after induction was resuspended in the appropriate buffer containing PLP at a 1:25 volume ratio (1 mL of the resuspension buffer per 25 mL of cell suspension) and sonicated (Soniprep 150 sonicator, MSE Sanyo Japan) on ice using 10 cycles of 10 s on, 10 s off at 10 Watts. The sonicated suspension was centrifuged at 12,000 rpm at 4 °C for 45 min to obtain the clarified lysate. The total protein concentration was determined using the Bradford method. TAm concentrations in the crude lysates were determined as 8.8 mg/mL *Cv*-

TAm, 8.0 mg/mL *Rh*-TAm, 9.6 mg/mL *Mv*-TAm, 6.4 mg/mL pQR2189,[1] 4.8 mg/mL pQR2191,[1] and 8.0 mg/mL pQR2208.[1]

**Colorimetric screening.** The assay was performed in a 96 well-plate with a total volume of 200 εL containing 2-(4-nitrophenyl)ethan-1-amine hydrochloride **5** (25 mM) as amine donor, amine acceptor (10 mM), PLP (0.5 mM) and potassium phosphate buffer (100 mM, pH 8.0). The reaction was started by the addition of TAm clarified lysate (20 εL) containing the overexpressed TAm and the reaction was incubated at 30 °C and 500 rpm for 24 h. A positive control was performed using benzaldehyde or pyruvate as the amine acceptor as specified. Two negative controls were also performed, one without amine acceptor and another without enzyme. An orange/red coloration indicated that the TAms were active towards the selected aldehydes.

**MBA screening.** The assay was performed in an Eppendorf tube (500 εL total volume) containing (*R*)- or (*S*)-MBA (20 mM), PLP (2 mM), potassium phosphate buffer (100 mM, pH 8.0), amine acceptor (5 mM) and crude cell lysate (30 εL) containing the overexpressed TAm. After incubation at 30 °C and 350 rpm for the time specified, the reaction was stopped by the addition of 0.1% trifluoroacetic acid (TFA) in water (100 εL). Denatured protein was removed by centrifugation (4 °C, 12000 rpm, 10 min) and the supernatant diluted with water and analysed by analytical HPLC using an ACE 5-C18 300 column (150 × 4.6 mm) with UV detection at 250 nm. The concentration of acetophenone produced was determined using a linear gradient 15%ï 72% over 10 minutes at 1 mL min$^{ï1}$ (A = water with 0.1% of TFA and B = acetonitrile) with subtraction of a negative control without amine acceptor for all substrates. The acetophenone (ACP) produced eluted at a retention time of 8.8 min. Results were verified in duplicate or triplicate.

**Isopropylamine assay.** The assay was performed in an Eppendorf tube containing isopropylamine (250 mM, pH 8), PLP (2 mM), potassium phosphate buffer (100 mM, pH 8), amine acceptor (25 mM) and crude cell lysate (30 εL) containing the overexpressed TAm in a final volume of 500 εL. After incubation at 37 °C and 400 rpm, the reaction was stopped by the addition of 1% TFA in water (100 εL). Denatured protein was removed by centrifugation (4 °C, 13000 rpm, 10 min) and the supernatant analysed by analytical HPLC after derivatization with 9-fluorenylmethyl chloroformate (Fmoc-Cl).

**Derivatization with Fmoc-Cl and analytical HPLC.** 25 εL of reaction sample was diluted with 5 εL of milliQ water or quenched with 1% TFA and mixed with 70 εL of sodium borate buffer solution (7 mM, pH 8) and 70 εL of Fmoc-Cl (40 mM in acetonitrile), and then incubated at r.t. for 5 min. After this time the reaction was stopped by adding 80 εL of L-alanine (100 mM in water:acetonitrile 2:1). After 1 min the reaction mixture was centrifuged (25 °C, 13000 rpm, 10 min) and the supernatant was analysed by HPLC using an ACE 5-C18 column (150 × 4.6 mm). The mobile phase A consisted of trifluoroacetic acid 0.1% in MilliQ water and the mobile phase B was trifluoroacetic acid 0.1% in acetonitrile 80% and MilliQ water 20%. The gradient elution was performed from 0 to 55% of phase B and run at 0.8 mL/min for 10 min at 30 °C for **3a** and from 40 to 60% of phase B and run at 0.8 mL/min for 20 min at 30 °C **3b** and for 28 min for **3c** and **3d**. The eluted products were detected at 360 nm and quantitative analysis was performed by the integration of peak areas using the external standard method (standard concentrations from 5 mM to 25 mM).

## II. Analytical HPLC chromatograms

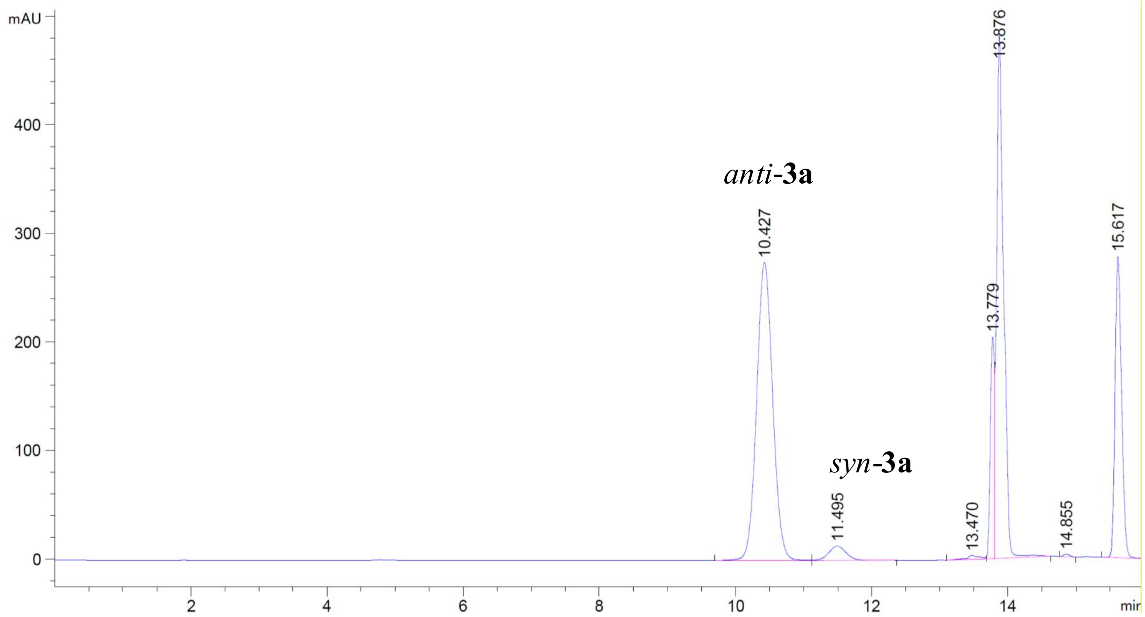


**Figure S1.** Typical HPLC chromatogram of the Fmoc-derivatives of *anti*-**3a** and *syn*-**3a**. The retention times for *anti*-**3b** and *syn*-**3b** were the same.

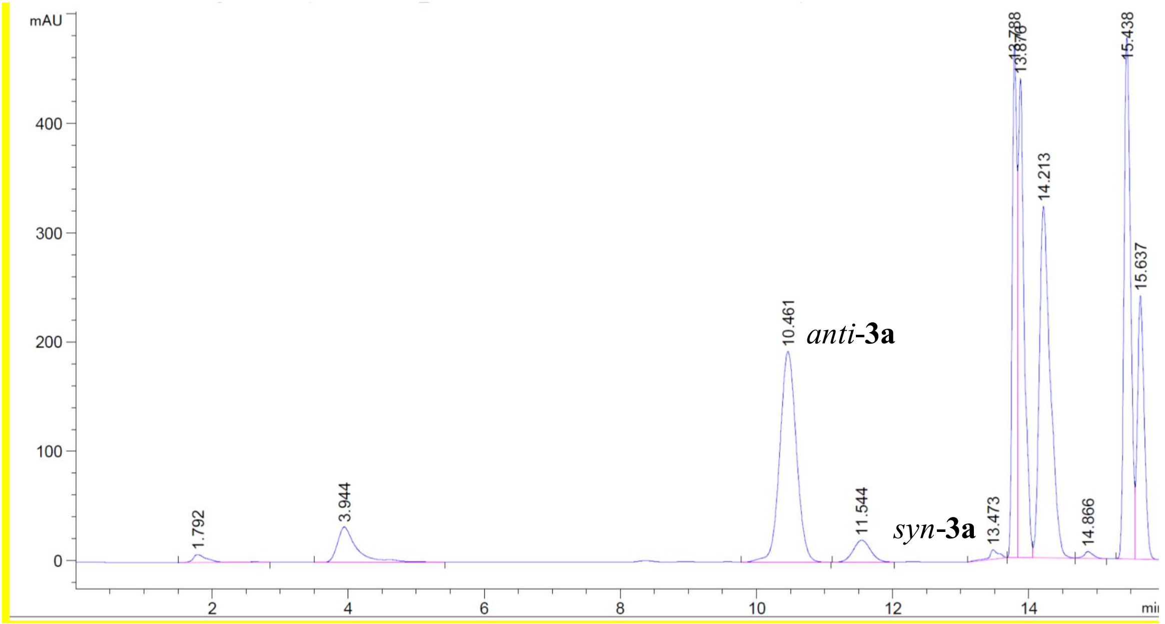


**Figure S1a.** Typical HPLC chromatogram of the biocatalytic reactions on **2a** after Fmoc-derivatization. Retention times for the products *anti*-**3a** and *syn*-**3a** are labelled. The retention times for *anti*-**3b** and *syn*-**3b** were identical.

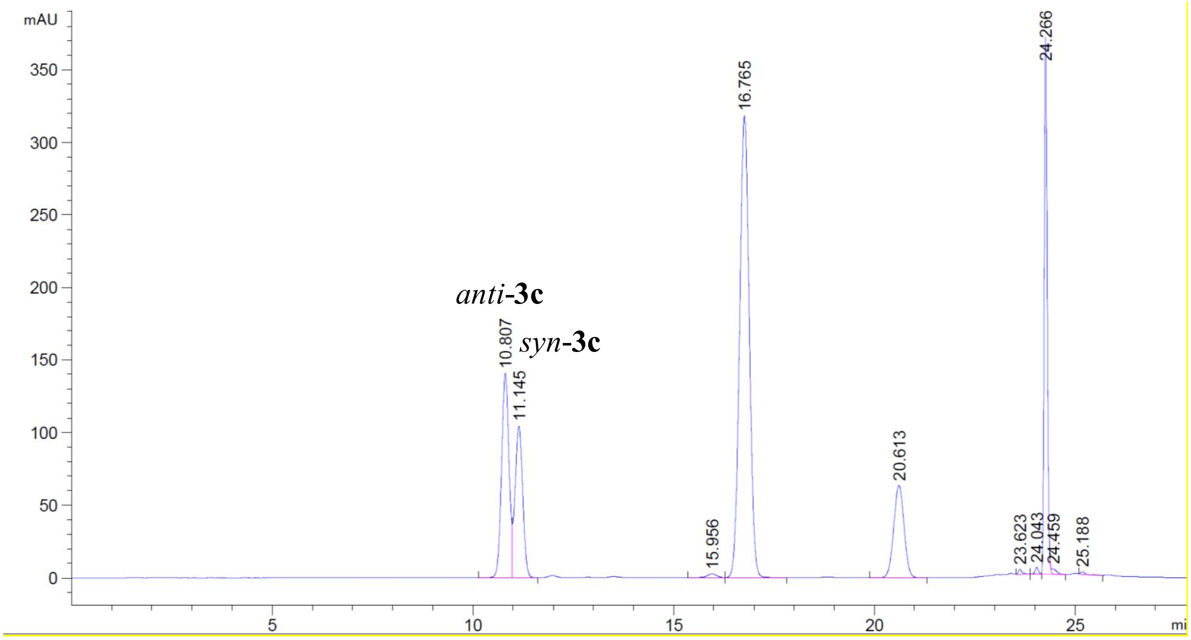


**Figure S2.** Typical HPLC chromatogram of the Fmoc-derivatives of *anti*-**3c** and *syn*-**3c**.

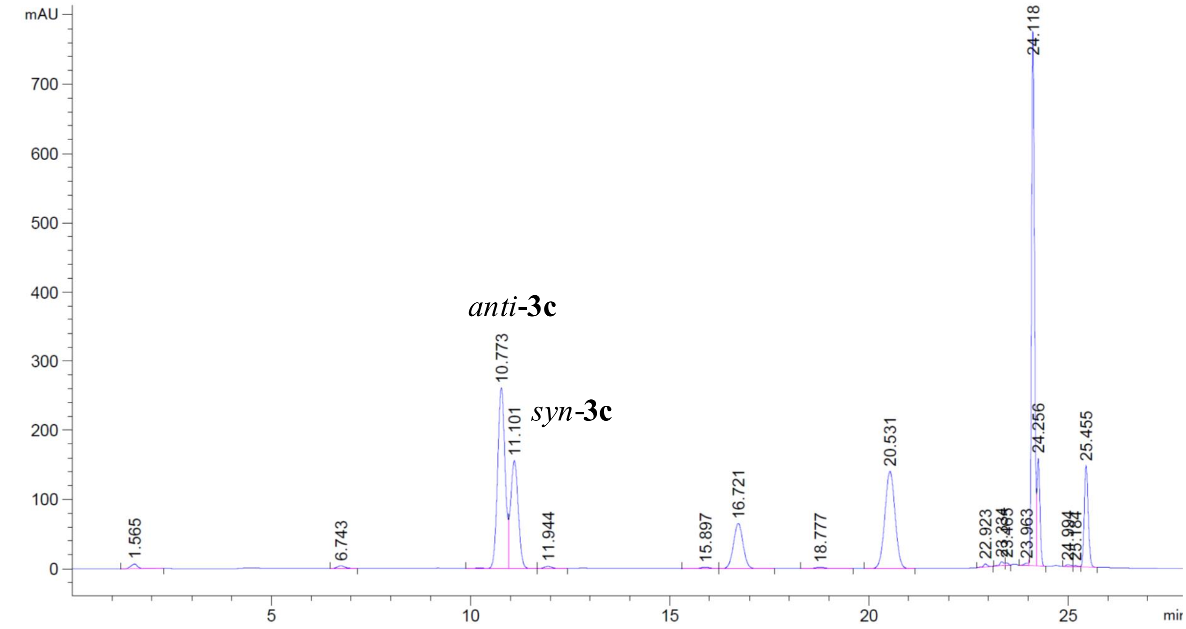


**Figure S2a.** Typical HPLC chromatogram of the biocatalytic reactions on **2c** after Fmoc-derivatization. Retention times for the products *anti*-**3c** and *syn*-**3c** are labelled.

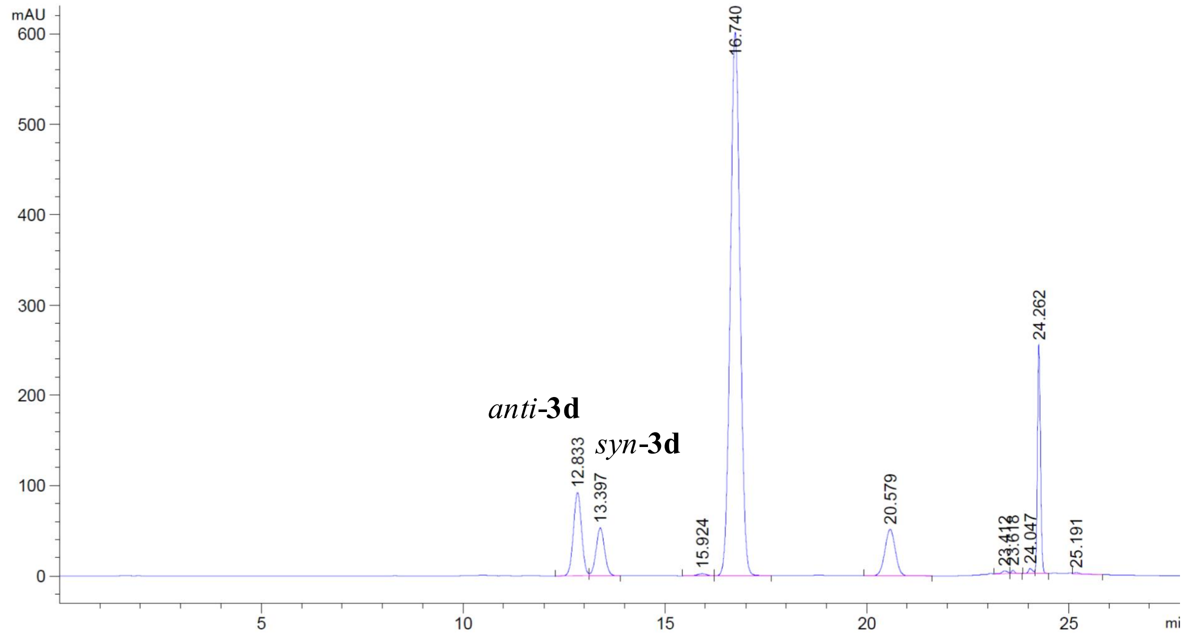


**Figure S3.** Typical HPLC chromatogram of the Fmoc-derivatives of *anti*-**3d** and *syn*-**3d**.

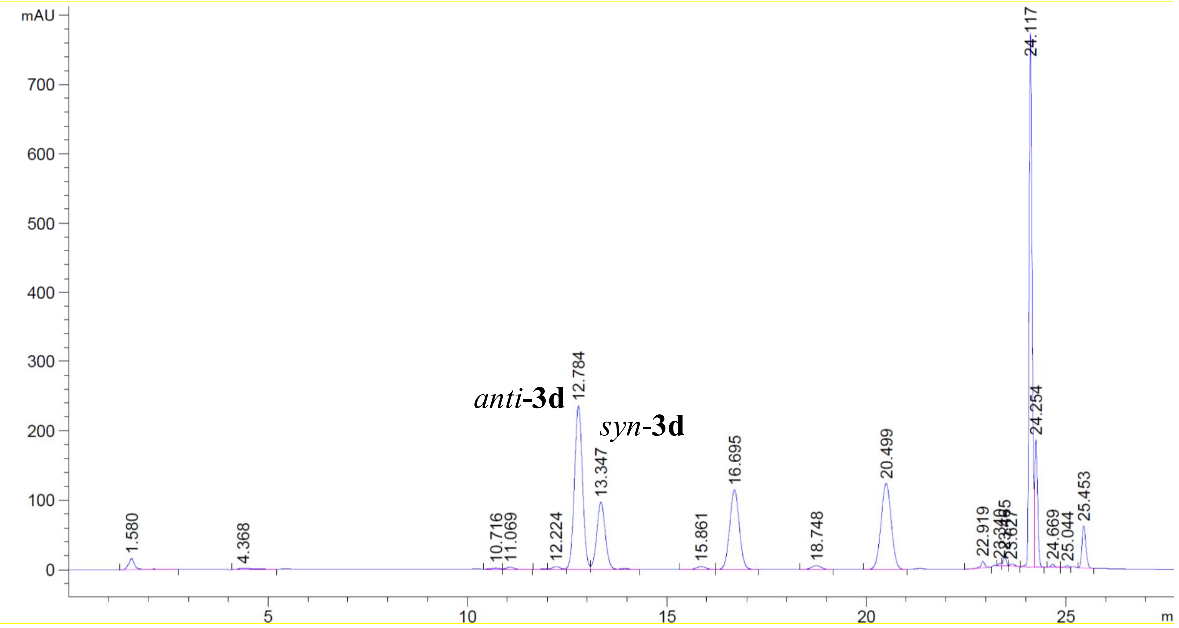


**Figure S3a.** Typical HPLC chromatogram of the biocatalytic reactions on **2d** after FMOC-derivatization. Retention times for the products *anti*-**3d** and *syn*-**3d** are labelled.

## III. Supplementary Data and Figures

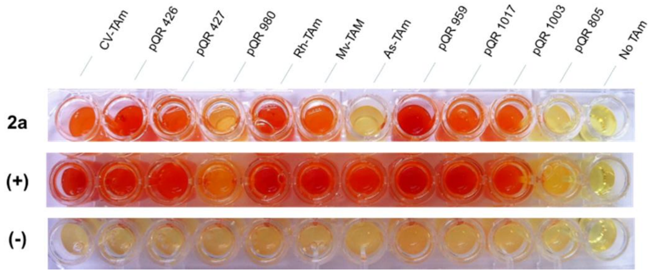


**Figure S4** Preliminary colorimetric assays on compound **2a** showed high acceptance for almost all the 11 selected TAms. They are *Chromobacterium violaceum* TAm (*Cv*-TAm),[2] *Pseudomonas aeruginosa* TAm (*Pa*-TAm pQR426),[2] *Pseudomonas putida* TAm (*Pp1*-TAm pQR427),[4] *Dienococcus geothermalis* TAm (*Dg*-TAm pQR980),[5] *Rhodobacter sphaeroides* TAm (*Rh*-TAm),[6] *Mycobacterium vanbaalenii* TAm (*Mv*-TAm),[6] *Arthrobacter sp.* variant ArRMut11 (ArRMut11),[7] *Pseudomonas putida* TAm (*Pp2*-TAm pQR959),[8] *Rhosospirillum rubrum* TAm (*Rr*-TAm pQR1017),[8] *Vibrio fluvialis* TAm (*Vf*-TAm),[9] *Saccharopolyspora erythraea* TAm (*Se*-TAm pQR805).[10] The assay was repeated in duplicate using 2-(4-nitrophenyl)ethan-1-amine **5** as amine donor: **5** (25 mM), **2a** (10 mM), PLP (0.5 mM), TRIS-HCl buffer pH 8.0 (50 mM) and enzyme clarified lysate (20 uL), 24 h, 30 °C, 500 rpm. Benzaldehyde was used as a positive control (+) and buffer as a negative control (ī ).

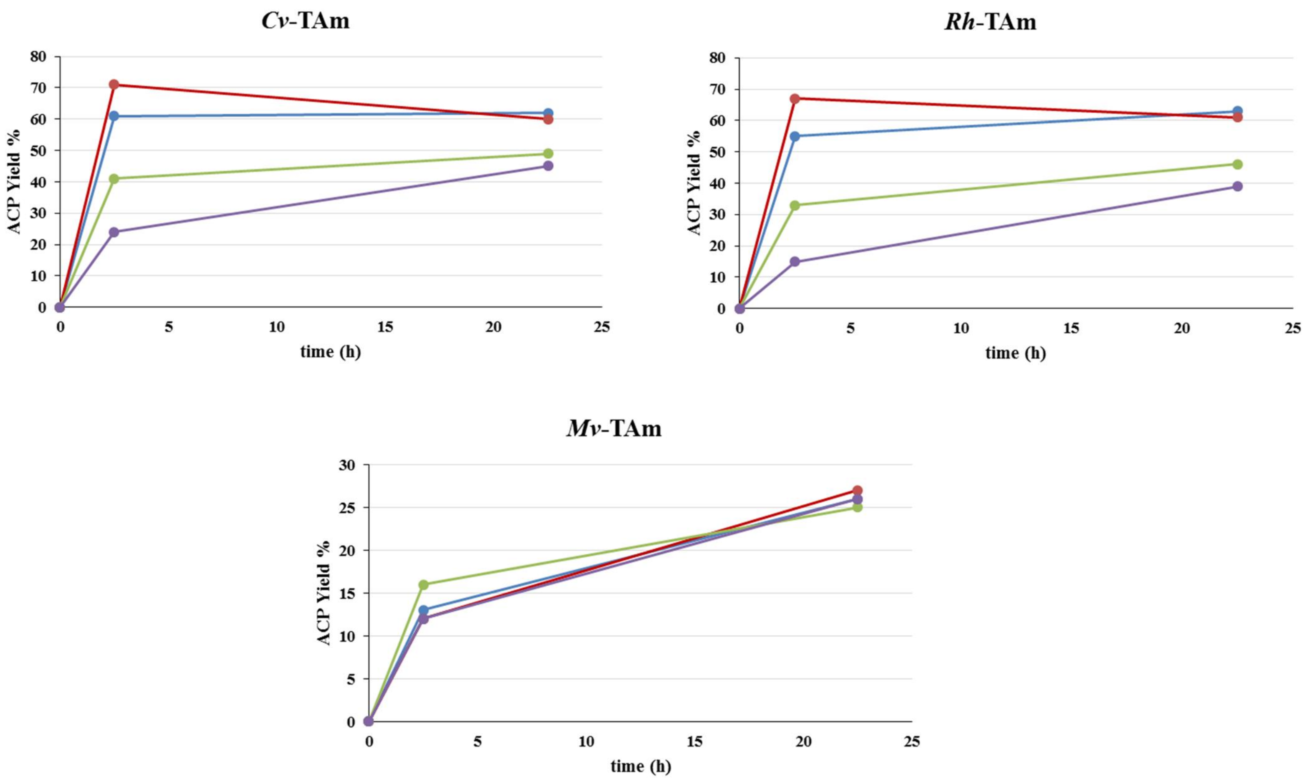


**Figure S5.** Time course analysis (2 h and 24 h) towards **2a-d** (5 mM) using 20 mM (*S*)-MBA (*Cv*-TAm and *Rh*-TAm) or 20 mM (*R*)-MBA (*Mv*-TAm) as amine donors, enzyme clarified lysate (1 mg/mL total protein), 24 h, 30 °C, 500 rpm. Blue (**2a**), Red (**2b**), Green (**2c**), and Purple (**2d**). The product acetophenone (ACP) was detected by HPLC analysis at 254 nm and used to determine % conversions. Background levels of acetophenone production were subtracted from the assay results in control reactions. All the reactions were performed in duplicate and average values are reported.

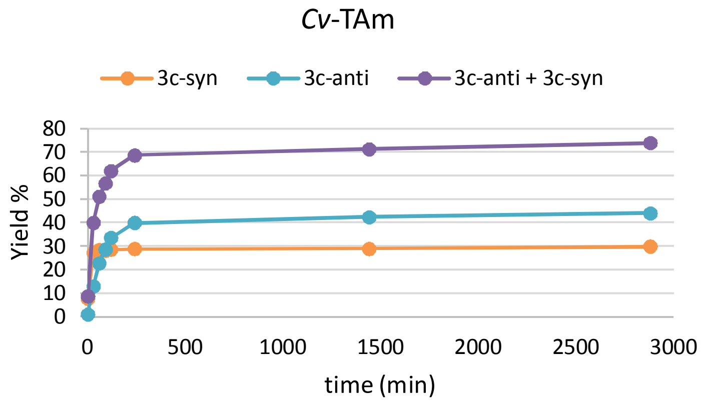


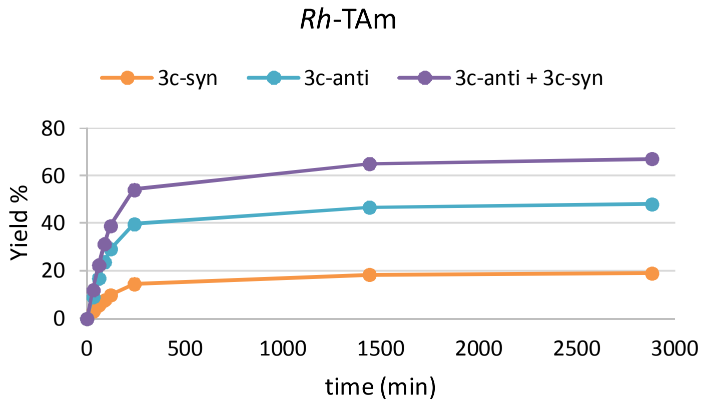


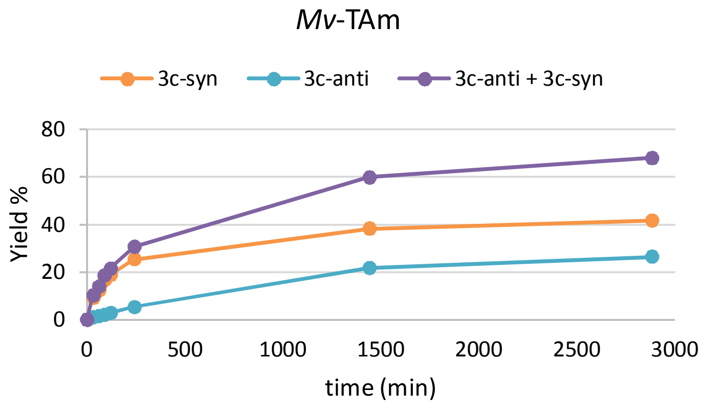


**Figure S6.** IPA Time course analysis towards **3c**. Screening results for substrate **3c** (25 mM) using 250 mM IPA as amine donors, enzyme clarified lysate (1 mg/mL), 24 h, 37 °C, 500 rpm. The product was detected by HPLC analysis after derivatization with Fmoc-Cl following the reported procedure in the experimental section and used to determine % yields.

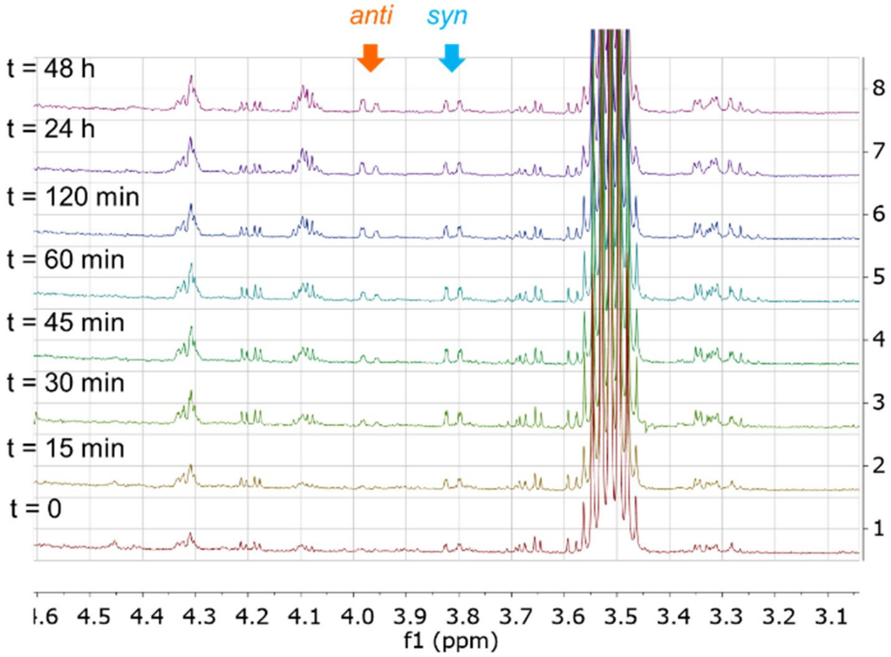


**Figure S7.** Time course analysis experiment using $^{1}$H NMR spectroscopy over 48 h using **2c** as starting material and *Cv*-TAm. **2c** (37.5 mg, 0.25 mmol) was dissolved in 3.45 ml of potassium phosphate buffer (50 mM, pH 8, containing 2 mM PLP) and IPA 1 M (1.250 mL, freshly prepared in potassium phosphate buffer, pH adjusted to 8) was added. Finally, 0.3 mL of *Cv*-TAm were added and the solution stirred at 37 °C and 250 rpm in a shake flask incubator. Aliquots (0.5 ml) were taken at different time points and quenched with 0.1 mL of HCl 1% and freeze dried. The residue was then dissolved in $D_2O$ and $^{1}$H NMR recorded. The signals at 3.8 ppm and 4.0 ppm are characteristic for *syn*-**3c** and *anti*-**3c** respectively.

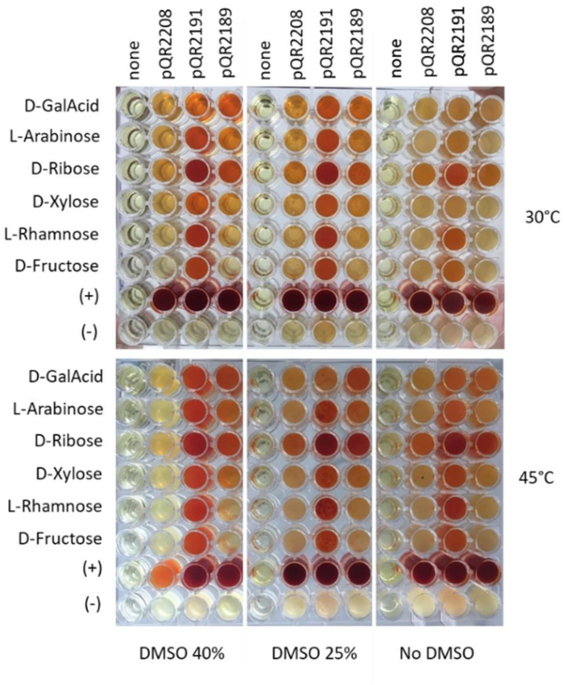


**Figure S8.** Colorimetric assay on selected sugars (10 mM), using **5** as the amine donor (25 mM), DMSO (25% or 40% v/v) were indicated, PLP (0.5 mM), potassium phosphate buffer pH 8.0 (100 mM) and enzyme clarified lysate (1 mg/mL), 24 h, 350 rpm. Pyruvate was used as a positive control (+) and buffer as a negative control (-). Reactions were carried out at different pHs (6.5, 8, and 9.5) and temperatures (30 °C and 45 °C).

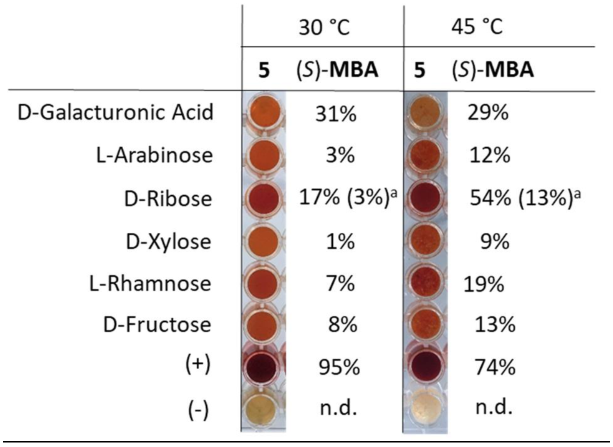


**Figure S9.** Colorimetric assay and conversions on selected sugars withpQR2191, using **5** or (*S*)-**MBA** as the amine donor, potassium phosphate buffer at pH 8.0, 24 h. Pyruvate was used as a positive control (+) and buffer as a negative control (-). Reactions were carried out at 30 °C or 45 °C as specified. [a] Control reaction without DMSO. It is noteworthy that the selected TAm pQR2191 performs well in 25% DMSO at 30 °C, as shown from the positive control with pyruvate (+), while the yield slightly decreased at 45 °C. Conversely, when sugars were used as substrates an increase in the conversion yields was observed at the higher temperature. Interestingly, the control reaction for D-ribose without DMSO showed lower conversion yields at both 30 °C or 45 °C. The conversion yields of D-ribose with pQR2191 also dropped when MeOH was used instead of DMSO.

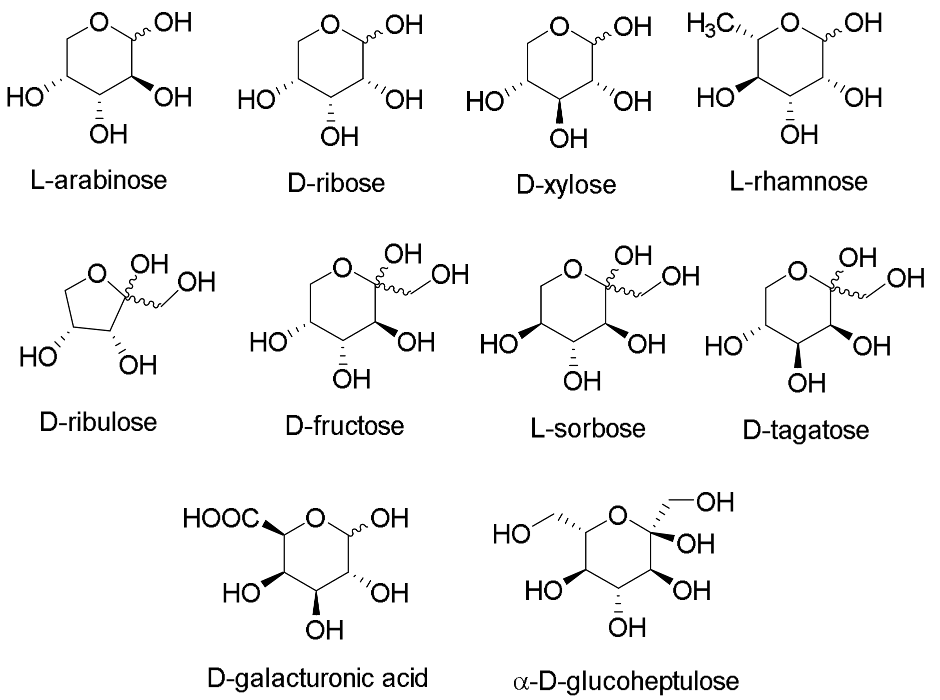


**Figure S10.** Structures of selected sugars. Only the pyranose form is shown (where possible).

## IV. Chemical syntheses and preparative scale biocatalytic reactions

### General procedure 1

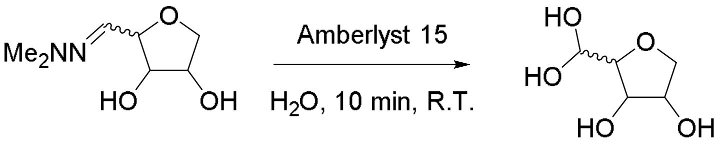


Amberlyst 15 (0.5 g/mmol) was added to a solution of hydrazone in water (C = 0.2 mol/L). The reaction was stirred at room temperature for 10 min before filtration on a sinter funnel (porosity 3). The resulting mixture was concentrated under vacuum and the resulting thick oil was lyophilised to afford the hydrate as a white foam.

**(2*S*,3*S*,4*S*)-2-(Dihydroxymethyl)tetrahydrofuran-3,4-diol (*anti*-2a) and (2*R*,3*S*,4*S*)-2-(dihydroxymethyl)tetrahydrofuran-3,4-diol (*syn*-2a)**

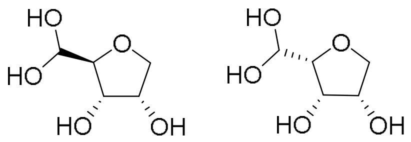


| **Ratio** | **80** | **20** |
|---|---|---|

The THF-hydrate was obtained following the **General procedure 1** using Amberlyst 15 (7 g) and THF-hydrazone **1a** (2.41 g, 13.8 mmol, *d.r.* = 50:50 (*anti:syn*)). After lyophilisation, the hydrate was isolated as a very hygroscopic white foam (1.59 g, 77%, *d.r.* = 80:20 (*anti:syn*)); $^{1}$H NMR (600 MHz, $D_2O$) ŭ = 5.16 (br d, *J* = 7.2, 1H, C*H*(OD)$_{2\text{-}syn}$), 5.04 (br d, *J* = 4.7, 1H, C*H*(OD)$_{2\text{-}anti}$), 4.47 (td, *J* = 7.6, 4.6, 1H, CH$_2$C*H*$_{\text{-}syn}$), 4.3ï 4.26 (m, 2H, CH$_2$C*H*$_{anti}$, OCH$_2$CHC*H*$_{syn}$), 4.22 (br t, *J* = 6.2, 5.0, 1H, OCH$_2$CHC*H*$_{anti}$), 4.05ï 4.02 (m, 2H, C*H*Hô$_{anti}$, C*H*Hô$_{syn}$), 3.81 (dd, *J* = 10.0, 3.2, 1H, CH*H*ô$_{anti}$), 3.78 (dd, *J* = 7.2, 3.7, 1H, CH(OD)$_2$C*H*$_{syn}$), 3.74 (dd, *J* = 6.2, 4.7, 1H, CH(OD)$_2$C*H*$_{anti}$), 3.71 (dd, *J* = 8.6, 7.6, 1H, CH*H*ô$_{syn}$); $^{13}$C{$^{1}$H} NMR (150 MHz, $D_2O$ with MeOH standard) ŭ = 89.9 (*C*H(OD)$_{2\text{-}anti}$), 88.7 (*C*H(OD)$_{2\text{-}syn}$), 83.7 (CH(OD)$_2$*C*H$_{anti}$), 82.7 (CH(OD)$_2$*C*H$_{syn}$), 72.4 (OCH$_2$*C*H$_{anti}$), 72.2 (O*C*H$_{2\text{-}anti}$), 71.3 (OCH$_2$CH*C*H$_{anti}$), 71.3 (OCH$_2$CH*C*H$_{syn}$), 71.0 (OCH$_2$*C*H$_{syn}$), 70.3 (O*C*H$_{2\text{-}syn}$). Data in agreement with that previously reported.[11]

**(2*R*,3*R*,4*R*)-2-(Dihydroxymethyl)tetrahydrofuran-3,4-diol (*anti*-2b) and (2*S*,3*R*,4*R*)-2-(dihydroxymethyl)tetrahydrofuran-3,4-diol (*syn*-2b)**

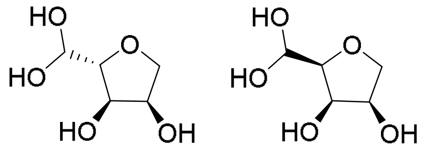

**Ratio 90 10**

The THF-hydrate was obtained following the **General procedure 1** using Amberlyst 15 (7 g) and THF-hydrazone **1b** (2.22 g, 12.7 mmol, *d.r.* = 80:20 (*anti:syn*)). After lyophilisation, the hydrate **2b** was isolated as a hygroscopic white foam (1.54 g, 79%, *d.r.* = 90:10 (*anti:syn*)). $^{1}$H NMR (600 MHz, $D_2O$) ŭ = 5.09 (br d, *J* = 7.3, 1H, C*H*(OD)$_{2\text{-}syn}$), 4.97 (d, *J* = 4.8, 1H, C*H*(OD)$_{2\text{-}anti}$), 4.42 (td, *J* = 7.4, 4.6, 1H, $OCH_2$C*H*$_{syn}$), 4.24ï 4.20 (m, 2H, $OCH_2$C*H*$_{anti}$, $CH_2$CHC*H*$_{syn}$), 4.19ï 4.15 (1H, m, $CH_2$CHC*H*$_{anti}$), 4.00ï 3.94 (2H, m, C*H*Hô$_{anti}$, C*H*Hô$_{syn,}$), 3.75 (dd, *J* = 10.0, 3.2, 1H, CH*H*ô$_{anti}$), 3.72 (dd, *J* = 7.3, 3.7, 1H, CH(OD)$_2$C*H*$_{syn}$), 3.68 (dd, *J* = 6.1, 4.8, 1H, CH(OD)$_2$C*H*$_{anti}$), 3.66ï 3.61 (m, 1H, CH*H*ô$_{syn}$); $^{13}$C{$^{1}$H} NMR ŭ = (150 MHz, $D_2O$) 90.4 (*C*H(OD)$_{2\text{-}anti}$), 89.2 (*C*H(OD)$_{2\text{-}syn}$), 84.1 (CH(OD)$_2$*C*H$_{anti}$), 83.3 (CH(OD)$_2$*C*H$_{syn}$), 73.0 ($OCH_2$*C*H$_{anti}$), 72.7 (O*C*H$_{2\text{-}anti}$), 71.9 ($CH_2$CH*C*H$_{anti}$), 71.8 ($CH_2$CH*C*H$_{syn}$), 71.5 ($OCH_2$*C*H$_{syn}$), 70.7 (O*C*H$_{2\text{-}syn}$); Characterization data was the same as for **2a**.

**(2*S*,3*S*,4*R*)-2-(Dihydroxymethyl)tetrahydrofuran-3,4-diol (*anti*-2c) and (2*R*,3*S*,4*R*)-2-(dihydroxymethyl)tetrahydrofuran-3,4-diol (*syn*-2c)**

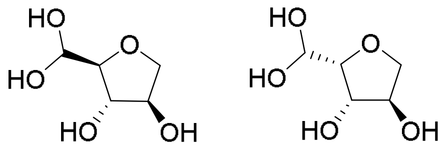

**Ratio 60 40**

The THF-hydrate was obtained following the **General procedure 1** using Amberlyst 15 (7 g) and THF-hydrazone **1c** (2.12 g, 12.1 mmol, *d.r.* = 55:45 (*anti:syn*)). After lyophilisation, the hydrate **2c** was isolated as a hygroscopic white foam (1.66 g, 91%, *d.r.* = 60:40 (*anti:syn*)). $^{1}$H NMR (600 MHz, $D_2O$) ŭ = 5.07 (br d, *J* = 7.5, 1H, C*H*(OD)$_{2\text{-}syn}$), 5.00 (d, *J* = 6.5, 1H, C*H*(OD)$_{2\text{-}anti}$), 4.26 (br d, *J* = 4.0, 1H, $OCH_2$C*H*$_{syn}$), 4.19ï 4.17 (m, 1H, $OCH_2$C*H*$_{anti}$), 4.16 (br d, *J* = 4.0, 1H, $CH_2$CHC*H*$_{syn}$), 4.15ï 4.11 (m, 2H, $CH_2$CHC*H*$_{anti}$, OC*H*Hô$_{syn}$), 3.97 (dd, *J* = 10.2, 4.0, 1H,

OC*H*Hô*anti*), 3.86ï 3.81 (m, 2H, OCH*H'anti*, CH(OD)$_2$C*H*$_{syn}$), 3.73 (br d, *J* = 10.2, 1H, OCH*H*ô*syn*), 3.62 (dd, *J* = 6.5, 3.0, 1H, CH(OD)$_2$C*H*$_{anti}$); $^{13}$C{$^1$H} NMR (150 MHz, $D_2O$ with MeOH standard) ŭ = 89.6 (*C*H(OD)$_{2\text{-}anti}$), 88.6 (*C*H(OD)$_{2\text{-}syn}$), 87.7 (CH(OD)$_2$*C*H$_{anti}$), 82.1 (CH(OD)$_2$*C*H$_{syn}$), 78.1 (OCH$_2$CH*C*H$_{anti}$), 76.9 (CH$_2$*C*H$_{anti}$), 76.6 (CH$_2$*C*H$_{syn}$), 75.9 (OCH$_2$CH*C*H$_{syn}$), 73.4 (O*C*H$_{2\text{-}syn}$), 73.2 (O*C*H$_{2\text{-}anti}$); FT-IR (ATR) *υ*/cm$^{ï1}$ = 3324 br, 2934, 1421; HRMS (ES-) found [M-$H_2O$+Cl]$^-$ 167.0112; $C_5H_8O_4Cl$ requires 167.0111.

**(2*R*,3*R*,4*R*,5*S*)-2-(Dihydroxymethyl)-5-methyltetrahydrofuran-3,4-diol (*anti*-2d) and (2*S*,3*R*,4*R*,5*S*)-2-(dihydroxymethyl)-5-methyltetrahydrofuran-3,4-diol (*syn*-2d)**

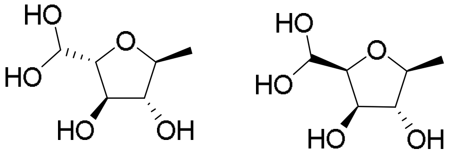


**Ratio 70 30**

The THF-hydrate was obtained following the **General procedure 1** using Amberlyst 15 (13 g) and the THF-hydrazone **1d** (5.00 g, 26.6 mmol, *d.r.* = 60:40 (*anti:syn*)). After lyophilisation, the THF-hydrate was isolated as a hygroscopic white foam (3.23 g, 81%, *d.r.* = 70:30 (*anti:syn*)). $^1$H NMR (600 MHz, $D_2O$) ŭ = 5.10 (br d, *J* = 7.2, 1H, C*H*(OD)$_{2\text{-}syn}$), 5.02 (d, *J* = 5.6, 1H, C*H*(OD)$_{2\text{-}anti}$), 4.12ï 4.09 (m, 2H, CH$_3$CHC*H*$_{syn}$, CH(OD)$_2$*C*H$_{anti}$), 3.89 (app. quint, *J* = 6.4, 1H, CH$_3$C*H*$_{anti}$), 3.84ï 3.78 (m, 2H, CH$_3$C*H*$_{syn}$, CH(OD)$_2$CHC*H*$_{syn}$), 3.78ï 3.72 (m, 2H, CH$_3$CHC*H*$_{anti}$, CH(OD)$_2$C*H*$_{syn}$), 3.67 (app. t, *J* = 5.7, 1H, CH(OD)$_2$CHC*H*$_{anti}$), 1.31 (d, *J* = 6.3, 3H, CH$_{3\text{-}syn}$), 1.24 (d, *J* = 6.4, 3H, CH$_{3\text{-}anti}$); $^{13}$C{$^1$H} NMR (150 MHz, $D_2O$ with MeOH standard) ŭ = 89.9 (*C*H(OD)$_{2\text{-}anti}$), 88.6 (*C*H(OD)$_{2\text{-}syn}$), 84.0 (CH(OD)$_2$*C*H$_{anti}$), 82.9 (CH(OD)$_2$*C*H$_{syn}$), 81.9 (CH$_3$CH*C*H$_{anti}$), 81.7 (O*C*H$_{3\text{-}syn}$), 78.8 (CH$_3$*C*H$_{anti}$), 77.7 (CH(OD)$_2$CH*C*H$_{anti}$), 77.5 (CH$_3$CH*C*H$_{syn}$), 18.5 (*C*H$_{3\text{-}syn}$), 17.7 (*C*H$_{3\text{-}anti}$); FT-IR (ATR) *υ*/cm$^{ï1}$ = 3319s br, 2926s, 1448m; HRMS (ES+) found [M+Na]$^+$ 187.0585; $C_6H_{12}O_5Na$ requires 187.0582.

**Boc-amines 4a-d synthesis.** Compounds **4a-d** were prepared following the General procedure 2.

**General procedure 2**

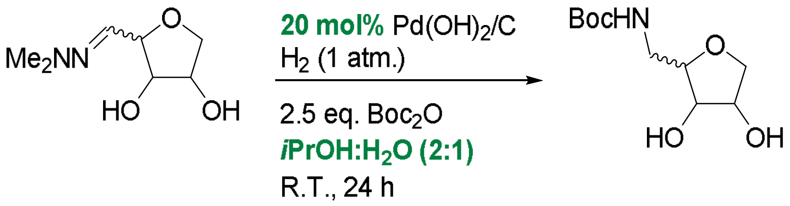


THF-hydrazone (1 eq.) was dissolved in a mixture of *i*PrOH:$H_2O$ (2:1, c = 0.05 mol/L). The solution was degassed with 3 vacuum/argon cycles before the addition of di-*tert*-butyl dicarbonate (2.5 eq.) and $Pd(OH)_2$ (20% on carbon, 0.02 eq.). The resulting mixture was then degassed with 3 vacuum/hydrogen cycles before hydrogen was bubbled for 5 minutes directly into the solution. The reaction was stirred under hydrogen atmosphere (1 atm.) at room temperature for 24 h before filtration through a pad of Celite. The filtrate was then concentrated under reduced pressure and the crude residue was purified by flash column chromatography using a gradient of petroleum ether 40-60:acetone (1:0 to 1:1) to give the Boc-amine.

***tert*-Butyl (((2*R*,3*S*,4*S*)-3,4-dihydroxytetrahydrofuran-2-yl)methyl)carbamate (*anti*-4a)**

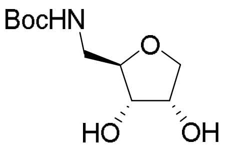


The protected amine was obtained by following **General procedure 2** using the THF-hydrazone *anti*-**1a** (250 mg, 1.44 mmo), di-*tert*-butyl dicarbonate (0.84 mL, 3.59 mmol) and $Pd(OH)_2$ (20% on carbon; 200 mg, 0.29 mmol) in a mixture of *i*PrOH:$H_2O$ (3 mL). After purification by column chromatography, the product was isolated as a colourless oil (125 mg, 38%, *anti* only); [□]□□ +34 (c 0.9, MeOH), lit [□]□□ +32 (c 1.0, MeOH),[12] $^{1}$H NMR (600 MHz, $CDCl_3$) 5.01 (s, 1H, N*H*), 4.24 (br s, 1H, $OCH_2$C*H*), 4.10 (dd, *J* = 10.1, 4.8, 1H, OC*H*Hô), 3.91ï 3.82 (m, 1H, $NCH_2$CHC*H*), 3.81ï 3.75 (m, 2H, OCH*H'*, $NCH_2$C*H*), 3.43ï 3.32 (m, 1H, NC*H*$_2$), 3.11 (s, 2H, O*H*), 2.01 (s, 2H, O*H*), 1.41 (s, 9H, 3 x C*H*$_3$); $^{13}$C{$^{1}$H} NMR (151 MHz, $CDCl_3$) 157.0 (*C*=O), 80.6 ($NCH_2$*C*H), 80.1 (*C*($CH_3$), 73.2 (O*C*$H_2$), 73.0 ($NCH_2$CH*C*H), 71.2 ($OCH_2$*C*H), 41.9 (N*C*$H_2$), 28.5 (*C*$H_3$) ppm;

FT-IR (ATR) $\upsilon$/cm$^{-1}$ = 3339, 2975, 2931, 1682, 1520; HRMS (CI) calcd for $C_{10}H_{20}NO_5$ $[M+H]^+$ 234.1336 found 234.1336.

***tert*-Butyl (((2*S*,3*R*,4*R*)-3,4-dihydroxytetrahydrofuran-2-yl)methyl)carbamate (*anti*-4b)**

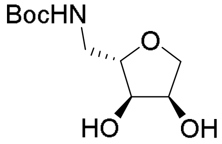


The protected amine was obtained by following **General procedure 2** using the THF-hydrazone *anti*-**1b** (50 mg, 0.29 mmol), di-*tert*-butyl dicarbonate (165 µL, 0.72 mmol) and $Pd(OH)_2$ (20% on carbon, 40 mg, 0.06 mmol) in a mixture of *i*PrOH:$H_2O$ (2 mL). After purification by column chromatography, the product was isolated as a colourless oil (41 mg, 61%, *anti* only); [[illegible]][illegible] -28 (c 0.8, MeOH); $^1H$ NMR (600 MHz, $CDCl_3$) 5.18 (br s, 1H, N*H*), 4.21 (br td, 1H, $OCH_2CH$), 4.10 (dd, *J* = 10.1, 4.8, 1H, OC*H*H'), 3.86–3.80 (m, 1H, $NCH_2CHCH$), 3.79–3.74 (m, 2H, OCH*H'*, $NCH_2CH$), 3.66 (br s, 2H, 2 x O*H*), 3.45–3.25 (m, 1H, $NCH_2$), 1.41 (s, 9H, 3 x $CH_3$); $^{13}C\{^1H\}$ NMR (151 MHz, $CDCl_3$) 157.0 (*C*=O), 80.6 ($NCH_2CH$), 80.1 ($C(CH_3)$), 73.2 ($OCH_2$), 73.0 ($NCH_2CHCH$), 71.2 ($OCH_2CH$), 41.9 ($NCH_2$), 28.5 ($CH_3$) ppm; FT-IR (ATR) $\upsilon$/cm$^{-1}$ = 3339, 2975, 2932, 1682, 1520; HRMS (CI) calcd for $C_{10}H_{20}NO_5$ $[M+H]^+$ 234.1336, found 234.1335.

***tert*-Butyl (((2*R*,3*S*,4*R*)-3,4-dihydroxytetrahydrofuran-2-yl)methyl)carbamate (*anti*-4c) and *tert*-butyl (((2*S*,3*S*,4*R*)-3,4-dihydroxytetrahydrofuran-2-yl)methyl)carbamate (*syn*-4c)**

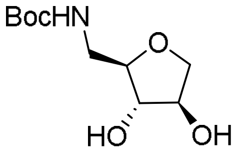


*anti*-**4c**

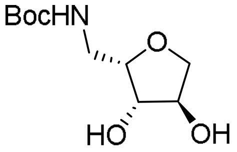


*syn*-**4c**

The protected amine was obtained by following **General procedure 2** using the THF-hydrazone **1c** (250 mg, 1.44 mmol, *d.r.* = 55:45 (*anti:syn*)),di-*tert*-butyl dicarbonate (0.84 mL, 3.59 mmol) and $Pd(OH)_2$ (20% on carbon, 200 mg, 0.29 mmol) in a mixture of *i*PrOH:$H_2O$ (3 mL). After purification by column chromatography, the product was isolated as a colourless oil (142 mg, 42%, *d.r.* = 55:45 (*anti:syn*)); $^1H$ NMR (600 MHz, $CDCl_3$) δ = 5.15 (br s, 1H, $NH_{anti}$), 5.07 (br s, 1H, $NH_{syn}$), 4.80 (s, 1H, $OH_{syn}$), 4.34 (br s, 1H, $OCH_2CH_{syn}$), 4.24 (br s, 1H, $OCH_2CH_{anti}$), 4.19 (dd, *J* = 9.8, 4.1, 1H, $OCHH'_{syn}$), 4.05–4.01 (m, 1H, $OCHH'_{anti}$), 3.97–3.90 (m, 1H, $NCH_2CHCH_{syn}$),

3.90–3.84 (m, 2H, $NCH_2CH_{syn}$, $NCH_2CHCH_{anti}$), 3.82 (br d, $J$ = 9.7, 1H, $OCHH'_{anti}$), 3.74 (br d, $J$ = 9.8, 1H, $NCH_2CH_{anti}$), 3.70 (br d, $J$ = 9.8, 1H, $OCHH'_{syn}$), 3.65–3.53 (m, 1H, $NCHH'_{syn}$), 3.45–3.35 (m, 2H, $NCH_{2\text{-}anti}$), 3.30 ( brs, 1H, $OH_{anti}$), 3.16 (dt, $J$ = 14.3, 4.7, 1H, $NCHH'_{syn}$), 2.67 (br s, 1H, $OH_{anti}$), 1.96 (br s, 1H, $OH_{syn}$), 1.43 (s, 18H, $C(CH_3)_{3\text{-}anti}$, $C(CH_3)_{3\text{-}syn}$; $^{13}C\{^1H\}$ NMR (151 MHz, $CDCl_3$) δ = 157.7 $(C{=}O)_{syn}$, 157.1 $(C{=}O)_{anti}$, 84.0 ($NCH_2CH_{anti}$), 80.86 ($NCH_2CHCH_{anti}$), 80.83 ($C(CH_3)_3$), 80.2 ($C(CH_3)_3$), 79.4 ($NCH_2CH_{syn}$), 77.7 ($OCH_2CH_{anti}$), 77.3 ($OCH_2CH_{syn}$ with $CDCl_3$), 76.3 ($NCH_2CHCH_{syn}$), 73.9 ($OCH_{2\text{-}syn}$), 73.1 ($OCH_{2\text{-}anti}$), 42.1 ($NCH_{2\text{-}anti}$), 38.6 ($NCH_{2\text{-}syn}$), 28.5 ($C(CH_3)_{3\text{-}anti}$), 28.4 ($C(CH_3)_{3\text{-}syn}$) ppm; FT-IR (ATR) $\upsilon$/cm$^{-1}$ = 3332, 2976, 2932, 1679, 1522; HRMS (CI) calcd for $C_{10}H_{20}NO_5$ $[M+H]^+$ 234.1336 found 234.1334.

***tert*-Butyl-(((2*R*,3*R*,4*R*,5*S*)-3,4-dihydroxy-5-methyltetrahydrofuran-2-yl)methyl)carbamate (*anti*-4d) and *tert*-butyl-(((2*S*,3*R*,4*R*,5*S*)-3,4-dihydroxy-5-methyltetrahydrofuran-2-yl)methyl)carbamate (*syn*-4d)**

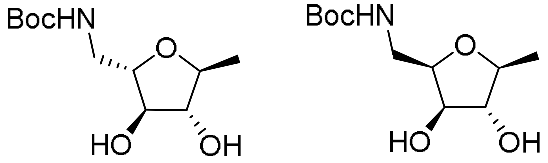


*anti*-**4d** *syn*-**4d**

The protected amine was obtained by following **General procedure 2** using the THF-hydrazone **1d** (290 mg, 1.50 mmol, *d.r.* = 60:40 (*anti:syn*)), di-*tert*-butyl dicarbonate (0.88 mL, 3.85 mmol) and $Pd(OH)_2$ (20% on carbon, 215 mg, 0.3 mmol) in a mixture of *i*PrOH:$H_2O$ (3 mL). After purification by column chromatography, the product was isolated as a colourless oil (117 mg, 30%, *d.r.* = 60:40 (*anti:syn*)); $^1H$ NMR (600 MHz, $CD_3OD$) δ = 3.95–3.91 (m, 1H, $NCH_2CHCH_{syn}$), 3.91–3.86 (m, 1H, $NCH_2CH_{syn}$), 3.85–3.76 (m, 3H, $CH_3CH_{anti}$, $CH_3CHCH_{anti}$, $NCH_2CH_{anti}$), 3.72–3.66 (m, 2H, $CH_3CH_{syn}$, $CH_3CHCH_{syn}$), 3.64 (br t, $J$ = 7.6, 1H, $NCH_2CHCH_{anti}$), 3.39 (dd, $J$ = 14.1, 6.4, 1H, $NCHH'_{syn}$), 3.29–3.20 (m, 2H, $NCH_{2\text{-}anti}$), 3.16 (dd, $J$ = 14.0, 6.3, 1H, $NCHH'_{syn}$), 1.44 (s, 18H, $C(CH_3)_{3\text{-}syn}$, $C(CH_3)_{3\text{-}anti}$), 1.28 (d, $J$ = 6.4, 3H, $CH_{3\text{-}syn}$), 1.26 (d, $J$ = 6.3, 3H, $CH_{3\text{-}anti}$); $^{13}C\{^1H\}$ NMR (151 MHz, $CD_3OD$) δ = 158.9 ($CO_{syn}$), 158.6 ($CO_{anti}$), 84.6 ($CH_3CH_{syn}$ or $CH_3CHCH_{syn}$), 84.1 ($NCH_2CHCH_{anti}$), 82.9 ($NCH_2CH_{anti}$ or $CH_3CHCH_{anti}$), 82.4 ($CH_3CH_{syn}$ or $CH_3CHCH_{syn}$), 81.3 ($NCH_2CH_{syn}$), 80.4 ($NCH_2CH_{anti}$ or $CH_3CHCH_{anti}$), 80.3 ($C(CH_3)_3$); 80.1 ($C(CH_3)_3$), 79.9 ($CH_3CH_{anti}$), 79.3 ($NCH_2CHCH_{syn}$), 43.5 ($NCH_{2\text{-}anti}$), 40.7 ($NCH_{2\text{-}syn}$), 28.73

(C($C$H$_3$)$_{3\text{-}anti}$), 28.70 (C($C$H$_3$)$_{3\text{-}syn}$) 19.5 ($C$H$_{3\text{-}syn}$), 19.1 ($C$H$_{3\text{-}anti}$) ppm; FT-IR (ATR) $\upsilon$/cm$^{ï1}$ = 3333, 2974, 2930, 1681, 1518; HRMS (CI) calcd for $C_{11}H_{20}NO_5$ $[M+H]^+$ 248.1492 found 248.1487.

**Amines 3a-d synthesis.** Compounds **3a-d** were prepared following the General procedure 3.

**General procedure 3**

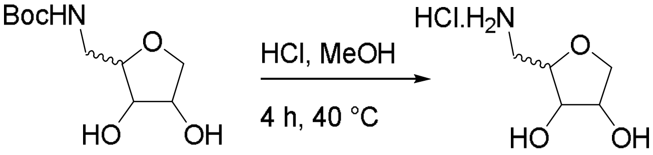


The protected amine was dissolved in a solution of HCl in MeOH (0.5 M) and heated at 40 °C for 4 hours. The reaction mixture was then evaporated to dryness. The residue was washed with $Et_2O$ and dried under high vacuum to give the ammonium salt.

**(2*R*,3*S*,4*S*)-2-(Aminomethyl)tetrahydrofuran-3,4-diol hydrochloride (3a)**

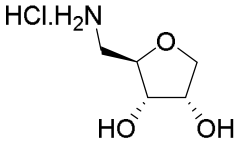


The amine hydrochloride salt was obtained by following the **General procedure 3** using the Boc-amine *anti*-**4a** (179 mg, 0.77 mmol, 1 eq.) and HCl in MeOH (4.6 mL, C = 0.5 mol/L, 2.30 mmol, 3 eq.). After evaporation of the volatiles under vacuum the amine salt was obtained as a white solid (129 mg, quant. *anti* only); mp = 173 - 175 °C (dec.); [⍰]⍰⍰⍰ + 55 (c 0.4, MeOH); $^1$H NMR (600 MHz, $CD_3OD$) ŭ = 4.17ï 4.13 (m, 1H, $OCH_2CH$), 4.10 (dd, $J$ = 9.9, 4.4, 1H, OC*H*Hô), 3.93ï 3.81 (m, 2H, $NCH_2CH$, $NCH_2CHCH$), 3.78 (dd, $J$ = 9.9, 2.3, 1H, OCH*H*ô), 3.21 (dd, $J$ = 13.0, 2.9, 1H, NC*H*Hô), 2.96 (dd, $J$ = 13.0, 8.7, 1H, NCH*H*ô); $^{13}$C{$^1$H} NMR (151 MHz, $CD_3OD$) ŭ = 78.7 ($NCH_2CH$), 75.3 ($NCH_2CHCH$), 74.6 ($OCH_2$), 72.3 ($OCH_2CH$), 43.2 ($NCH_2$) ppm; FT-IR (ATR) $\upsilon$/cm$^{ï1}$ = 3444, 3233, 3142, 3000, 2941, 2884, 1586, 1515, 1474, 1458; HRMS (ESI) calcd for $C_5H_{12}NO_3$ $[M\text{-}Cl]^+$ 134.0812, found 134.0811.

**(2*S*,3*R*,4*R*)-2-(Aminomethyl)tetrahydrofuran-3,4-diol hydrochloride (3b)**

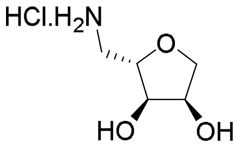

The amine hydrochloride salt was obtained by following the **General procedure 3** using the Boc-amine *anti*-**4b** (179 mg, 0.77 mmol) and HCl in MeOH (4.6 mL, c = 0.5 mol/L, 2.30 mmol). After evaporation of the volatiles under vacuum the amine salt was obtained as a white solid (129 mg, Quant. *anti* only); mp = 175 - 176 °C (dec.); [□]□□ ĭ 49 (c 0.5, MeOH), lit [□]□□ ĭ 57 (c 1.56, $H_2O$)[11]; $^1$H NMR (600 MHz, $CD_3OD$) ŭ = 4.17ĭ 4.13 (m, 1H, $OCH_2CH$), 4.10 (dd, *J* = 9.9, 4.6, 1H, OC*H*'H), 3.91ĭ 3.88 (m, 1H, $NCH_2CH$), 3.85 (dd, *J* = 7.7, 4.8, $NCH_2CHCH$), 3.78 (dd, *J* = 9.9, 2.4, 1H, OCH*H*') 3.22 (dd, *J* = 13.0, 2.9, 1H, NC*H*'H), 2.97 (dd, *J* = 13.0, 8.7, 1H, NCH*H*'); $^{13}$C{$^1$H} NMR (151 MHz, $CD_3OD$) ŭ = 78.7 ($NCH_2CH$), 75.3 ($NCH_2CHCH$), 74.6 ($OCH_2$), 72.3 ($OCH_2CH$), 43.2 ($NCH_2$); FT-IR (ATR) *υ*/cmĭ1 = 3445, 3234, 3144, 2999, 2940, 2913; 1587, 1515; HRMS (CI) calcd for $C_5H_{12}NO_3$ [M-Cl]$^+$ 134.0812 found 134.0811.

**(2*R*,3*S*,4*R*)-2-(Aminomethyl)tetrahydrofuran-3,4-diol hydrochloride (*anti*-3c) and (2*S*,3*S*,4*R*)-2-(aminomethyl)tetrahydrofuran-3,4-diol hydrochloride (*syn*-3c)**

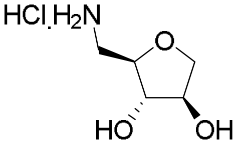

*anti*-**3c**

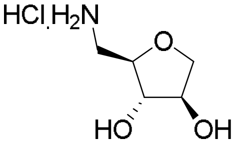

*syn*-**3c**

The amine hydrochloride salt was obtained by following the **General procedure 3** using the Boc-amine **4c** (152 mg, 0.65 mmol, 1 eq., *d.r.* = 59:41 (*anti:syn*)) and HCl in MeOH (4.0 mL, C = 0.5 mol/L, 1.95 mmol, 3 eq.). After evaporation of the volatiles under vacuum the amine salt was obtained as a colourless oil (109 mg, Quant., *d.r.* = 59:41 (*anti:syn*)); $^1$H NMR (600 MHz, $CD_3OD$) ŭ = 4.20ĭ 4.17 (m, 1H, $OCH_2CH_{syn}$), 4.17ĭ 4.10 (m, 4H, $OCH_2CH_{anti}$, $OCHH_{syn}$, $NCH_2CH_{syn}$, $NCH_2CHCH_{syn}$), 4.02 (dd, *J* = 9.7, 3.6, 1H, OC*H*Hǭnti), 3.97ĭ 3.91 (m, 2H, $NCH_2CH_{anti}$, $NCH_2CHCH_{anti}$), 3.89 (d, *J* = 9.7, 1H, OCH*H*'$_{anti}$), 3.72 (d, *J* = 8.7, 1H, $OCHH_{syn}$), 3.21 (br d, *J* = 4.7, 2H, $NCH_{2\text{-}syn}$), 3.15 (d, *J* =5.8, 2H, $NCH_{2\text{-}anti}$); $^{13}$C{$^1$H} NMR (151 MHz, $CD_3OD$) ŭ = 83.3

(NCH$_2$*C*H$_{anti}$), 80.7 (NCH$_2$CH*C*H$_{anti}$), 79.0 (*C*H$_{syn}$), 78.6 (*C*H$_{syn}$), 78.2 (OCH$_2$*C*H$_{anti}$), 77.1 (*C*H$_{syn}$), 75.6 (O*C*H$_{2\text{-}anti}$), 74.9 (O*C*H$_{2\text{-}syn}$), 42.7 (N*C*H$_{2\text{-}anti}$), 40.5 (N*C*H$_{2\text{-syn}}$) ppm. FT-IR (ATR) $\upsilon$/cm$^{ï1}$ = 3246, 2921, 1605, 1501, 1456; HRMS (ESI) calcd for $C_5H_{12}NO_3$ [M-Cl]$^+$ 134.0812 found 134.0812.

**(2*R*,3*R*,4*R*,5*S*)-2-(Aminomethyl)-5-methyltetrahydrofuran-3,4-diol hydrochloride (*anti*-3d) and (2*S*,3*R*,4*R*,5*S*)-2-(aminomethyl)-5-methyltetrahydrofuran-3,4-diol hydrochloride (*syn*-3d)**

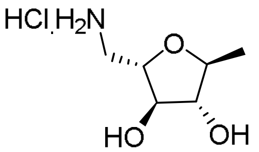

*anti*-**3d**

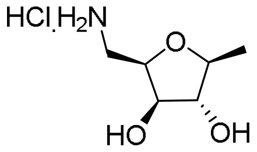

*syn*-**3d**

The amine hydrochloride salt was obtained by following the **General procedure 3** using the Boc-amine **4d** (120 mg, 0.48 mmol, *d.r.* = 60:40 (*anti:syn*)) and HCl in MeOH (3.0 mL, c = 0.5 mol/L, 1.45 mmol). After evaporation of the volatiles under vacuum the amine salt was obtained as a colourless oil (86 mg, 96%, *anti*:*syn* = 63:37); $^1$H NMR (600 MHz, CD$_3$OD) ŭ = 4.13 (m, 1H, NCH$_2$C*H*$_{syn}$), 4.09 (dd, *J* = 4.4, 2.0, 1H, NCH$_2$CHC*H*$_{syn}$), 3.99 (td, *J* = 8.7, 4.4, 1H, NCH$_2$C*H*$_{anti}$), 3.95 (br quint, *J* = 12.2, 1H, CH$_3$C*H*$_{anti}$), 3.86ï 3.82 (m, 1H, NCH$_2$CHC*H*$_{anti}$), 3.83ï 3.78 (m, 1H, CH$_3$C*H*$_{syn}$), 3.75 (m, 1H, CH$_3$CHC*H*$_{anti}$), 3.72 (dd, *J* = 3.8, 2.1, 1H, CH$_3$CHC*H*$_{syn}$), 3.24ï 3.18 (m, 2H, NC*H*$_{2\text{-}syn}$), 3.16 ï 3.06 (m, 2H, NC*H*$_{2\text{-}anti}$), 1.34 (d, *J* = 6.3, 3H, C*H*$_{3\text{-}syn}$), 1.32ï 1.28 (m, 3H, C*H*$_{3\text{-}anti}$) ; $^{13}$C{$^1$H} NMR (151 MHz, CD$_3$OD) ŭ = 84.7 (OCH*CH*$_{syn}$), 83.6 (OCH*CH*$_{anti}$), 82.9 (CH$_3$*C*H$_{syn}$), 81.4 (CH$_3$*C*H$_{anti}$), 80.8 (NCH$_2$*C*H, NCH$_2$CH*C*H$_{anti}$), 80.3 (NCH$_2$CH*C*H$_{syn}$), 77.3 (NCH$_2$*C*H$_{syn}$), 42.7 (N*C*H$_{2\text{-}anti}$), 40.6 (N*C*H$_{2\text{-}syn}$), 19.5 (*C*H$_{3\text{-}syn}$), 19.2 (*C*H$_{3\text{-}anti}$) ppm ; FT-IR (ATR) $\upsilon$/cm$^{ï1}$ = 3238, 2969, 2920, 1605, 1493, 1451; HRMS (ESI) calcd for $C_6H_{14}NO_3$ [M-Cl]$^+$ 148.0968 found 148.0968.

**General procedure 4:** Preparative scale biocatalytic reactions on substrates **2a-d**

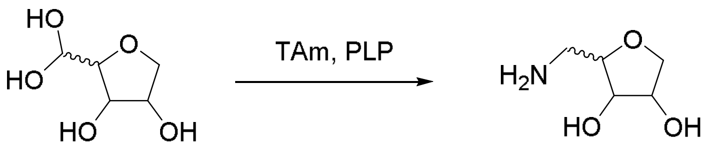


The enzymatic reactions were performed using a total volume of 20 mL or 40 mL, containing IPA (250 mM) as amine donor, and the aldehydes **2a-d** (50 mM, 1 mmol or 2 mmol) as amine acceptor, PLP (2 mM), potassium phosphate buffer pH 8.0 (50 mM), clarified cell lysate of *Cv*-TAm or *Rh*-TAm (1.5 or 3 ml) at 37 °C and 250 rpm for 24 h in a falcon tube. The reaction mixture was basified with NaOH to pH 12 and centrifuged. The supernatant was freeze dried to remove water and excess IPA and the yellow residue dissolved in methanol to prepare the Boc-amine following the **General procedure 3**. $Boc_2O$ (1.2 equiv.) was added at room temperature and the reaction mixture was stirred for 24 h. Methanol was removed under reduced pressure and the residue dissolved in EtOAc and filtered through celite. After evaporation to dryness, a pale yellow oil corresponding to the Boc-derived compound was obtained and further purified with silica column chromatography with a gradient of petroleum ether 40-60:acetone (1;0 to 1:1). The Boc deprotection was carried out in MeOH-HCl 0.5 M at 40 °C.

**(2*R*,3*S*,4*S*)-2-(Aminomethyl)tetrahydrofuran-3,4-diol hydrochloride (*anti*-3a) and (2*S*,3*S*,4*S*)-2-(aminomethyl)tetrahydrofuran-3,4-diol hydrochloride (*syn*-3a)**

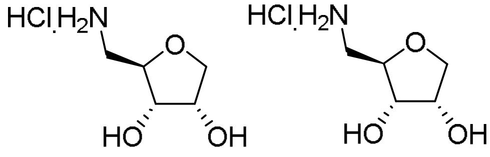

*anti*-**3a** *syn*-**3a**

The aldehyde **2a** (300 mg, 2.00 mmol) was subjected to the **General procedure 4** using *Rh*-TAm (3 mL) in a total volume of 40 mL, quenched with NaOH and the denatured protein removed by centrifugation. The supernatant was freeze dried and the residue dissolved in MeOH (8 mL). To this solution was added $Boc_2O$ (550 mg, 2.4 mmol) and the reaction was stirred at room temperature for 24 h. After work up as described in the **General procedure 3** the purification by column chromatography afforded compound **4a** as a pale yellow oil (330 mg, 71%, *anti:syn*

90:10). An aliquot (200 mg, 0.86 mmol) was deprotected in MeOH-HCl (0.5 M, 6 mL) for 16 h, and the solvent removed under reduced pressure to give the 2-(aminomethyl)tetrahydrofuran-3,4-diol hydrochloride **3a** (145 mg, quant., *anti:syn* 90:10). The characterisation data for the *anti* isomer was identical to **3a** synthesised above.

**(*2S,3R,4R*)-2-(Aminomethyl)tetrahydrofuran-3,4-diol hydrochloride (*anti*-3b) and (*2R,3R,4R*)-2-(aminomethyl)tetrahydrofuran-3,4-diol hydrochloride (*syn*-3b)**

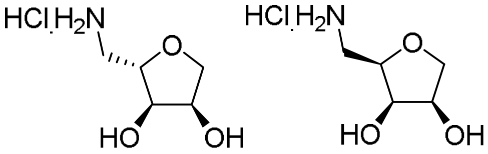


*anti*-**3b** *syn*-**3b**

The aldehyde **2b** (150 mg, 1.00 mmol was subjected to the **General procedure 4** using *Cv*-TAm (1.5 mL) in a total volume of 20 mL, quenched with NaOH and the denatured protein removed by centrifugation. The supernatant was freeze dried and the residue dissolved in MeOH (2 mL). To this solution was added $Boc_2O$ (275 mg, 1.2 mmol) and the reaction was stirred at room temperature for 24 h. After work up as described in the general procedure the purification by column chromatography afforded compound **4b** as a clear oil (177 mg, 76%, *anti:syn* 90:10). This was deprotected in MeOH-HCl (0.5 M, 3 mL) for 16 h, and the solvent removed under reduced pressure to give the 2-(aminomethyl)tetrahydrofuran-3,4-diol hydrochloride **3b** (127 mg, quant, *anti:syn* 90:10). The characterisation data for the *anti* isomer was identical to **3b** synthesised above.

**(2*R*,3*S*,4*R*)-2-(Aminomethyl)tetrahydrofuran-3,4-diol hydrochloride (*anti*-3c) and (2*S*,3*S*,4*R*)-2-(aminomethyl)tetrahydrofuran-3,4-diol hydrochloride (*syn*-3c)**

The aldehyde **2c** (150 mg, 1.00 mmol) was subjected to the **General procedure 4** using *Rh*-TAm (1.5 mL) in a total volume of 20 mL, quenched with NaOH and the denatured protein removed by centrifugation. The surnatant was freeze dried and the residue dissolved in MeOH (2 mL). To this solution was added $Boc_2O$ (275 mg, 1.2 mmol) and the reaction was stirred at room temperature for 24 h. After work up as described in the general procedure the purification by column chromatography afforded compound **4c** as a colourless oil (160 mg, 69%, *anti:syn* 55:45). An

aliquot (100 mg, 0.43 mmol) was deprotected in MeOH-HCl (0.5 M, 3 mL) for 16 h, and the solvent removed under reduced pressure to give the 2-(aminomethyl)tetrahydrofuran-3,4-diol hydrochloride **3c** (72 mg, quant, *anti:syn* 60:40). The characterisation data was identical to **3c** synthesised above.

**2*R*,3*R*,4*R*,5*S*)-2-(Aminomethyl)-5-methyltetrahydrofuran-3,4-diol hydrochloride (*anti*-3d) and (2*S*,3*R*,4*R*,5*S*)-2-(aminomethyl)-5-methyltetrahydrofuran-3,4-diol hydrochloride (*syn*-3d)**

The aldehyde **2d** (328 mg, 2.00 mmol) was subjected to the **General procedure 4** using *Rh*-TAm (3 mL) in a total volume of 40 mL, quenched with NaOH and the denatured protein removed by centrifugation. The supernatant was freeze dried and the residue dissolved in MeOH (8 mL). To this solution was added $Boc_2O$ (550 mg, 2.4 mmol) and the reaction was stirred at room temperature for 24 h. After work up as described in the general procedure the purification by column chromatography afforded compound **4d** as a colourless oil (290 mg, 58%, *anti:syn* 70:30). This was deprotected in MeOH-HCl (0.5 M, 8 mL) for 16 h, and the solvent removed under reduced pressure to give the 2-(aminomethyl)-5-methyltetrahydrofuran-3,4-diol hydrochloride **3d** (213 mg, quant, *anti:syn* 70:30). The characterisation data was identical to **3d** synthesised above.

**General procedure 5:** Preparative scale biocatalytic reactions on aldose sugar substrates (L-arabinose and D-xylose) with IPA

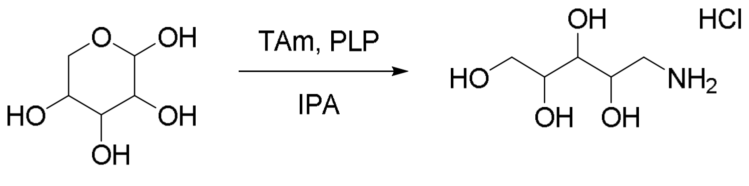


The enzymatic reactions were performed using a total volume of 30 mL, containing IPA (250 mM) as amine donor, sugar substrate (25 mM, 0.75 mmol), PLP (2 mM), potassium phosphate buffer pH 8.0 (50 mM), clarified cell lysate of *Rh*-TAm (2 mL) at 30 °C and 250 rpm. After 4 h, another aliquot of clarified cell lysate of *Rh*-TAm (2 mL) was added together with 5 mg of PLP. After 48 h the reaction mixture was quenched with MeOH and centrifuged (10 min, 4000 rpm). The

supernatant was basified to pH 12 with NaOH and concentrated to remove excess IPA. The yellow residue was redissolved in water (50 mL, pH adjusted to 8.0), loaded on to a Dowex 50WX8 ion-exchange resin, and washed several times with water (100 mL) to remove excess salt. The amine product was then eluted with 28% aq $NH_3$ (60 mL) and the eluent was concentrated under reduced pressure. The off-white residue obtained was resuspended in 1 M HCl and concentrated to dryness to give the amine salt as a single isomer.

**1-Amino-1-deoxy-L-arabinitol hydrochloride (7)**

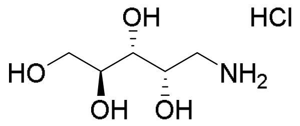


L-arabinose was subjected to the **General Procedure 5** and 1-amino-1-deoxy-L-arabinitol hydrochloride **7** was obtained as a solid (58.6 mg, 42%); $^1$H NMR (700 MHz, $D_2O$) ŭ = 4.22 (td, *J* = 6.5, 1.8, 1H, C*H*$CH_2$N), 3.89 (dd, *J* = 11.9, 2.9, 1H, CH*H*O), 3.80 (ddd, *J* = 8.9, 6.0, 2.9, 1H, C*H*$CH_2$O), 3.72 (dd, *J* = 11.9, 6.0, 1H, CH*H*CHOH), 3.60 (dd, *J* = 8.9, 1.8, 1H, CHC*H*CH), 3.24 (d, *J* = 6.5, 1H, CH*H*N); $^{13}$C NMR (176 MHz, $D_2O$ with MeOH standard) ŭ = 71.3 (*C*H$CH_2$OH), 70.7 (CH*C*HCH), 66.5 (*C*H$CH_2$N), 62.9 (*C*$H_2$OH), 42.7 (*C*$H_2$N) ppm; Mp 126 ± 2 °C (with decomposition); HRMS (ESI+) calcd for $C_5H_{14}NO_4$ $[M+H]^+$ 152.0917 found 152.0914. Characterisation data was in agreement with the literature.[13]

**1-Amino-1-deoxy-D-xylitol hydrochloride (8)**

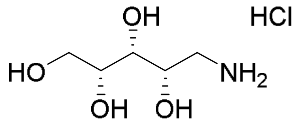


D-xylose was subjected to the **General Procedure 5** and 1-amino-1-deoxy-D-xylitol hydrochloride **8** was obtained as a solid (95% purity by $^1$H NMR - $NH_4Cl$ present) (117.5 mg, 79%). A batch of material was re-purified with the Dowex resin to give the hydrochloric salt for further NMR analysis; $^1$H NMR (700 MHz, $D_2O$) ŭ = 4.09 (dt, *J* = 9.3, 3.5, 1H, C*H*$CH_2$N), 3.88 (dd, *J* = 11.9, 2.9, 1H, C*H*$CH_2$O), 3.78 (dd, *J* = 11.9, 4.3, 1H, C*H*HOH), 3.73 ï 3.69 (m, 2H,

CH*H*OH and CHC*H*CH), 3.29 (dd, *J* = 13.1, 3.2, 1H, CH*H*N), 3.21 (dd, *J* = 13.1, 9.3, 1H, CH*H*N); $^{13}$C NMR (176 MHz, $D_2O$ with MeOH standard) ŭ = 71.6 (*C*HCH$_2$OH and CH*C*HCH), 67.9 (*C*HCH$_2$N), 62.5 (*C*H$_2$OH), 42.1 (*C*H$_2$N) ppm; Mp 110 ± 2 °C (with decomposition), HRMS (ESI+) calcd for $C_5H_{14}NO_4$ $[M+H]^+$ 152.0917 found 152.0914.

**Preparative scale biocatalytic reactions for D-fructose with IPA to give the 2-amino-2-deoxy-D-mannitol hydrochloride (9)**

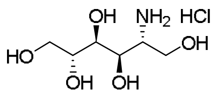


The enzymatic reaction was performed using a total volume of 30 mL, containing IPA (250 mM) as amine donor, and D-fructose (25 mM, 0.75 mmol), PLP (2 mM), potassium phosphate buffer pH 8.0 (50 mM), clarified cell lysate of *Mv*-TAm (3.6 mL) at 45 °C and 250 rpm for 48 h. The reaction mixture was quenched with MeOH and centrifuged (10 min, 4000 rpm). The supernatant was basified to pH 12 with NaOH and concentrated to remove excess IPA. The yellow residue was redissolved in water (50 mL, pH adjusted to 8.0), loaded on to a Dowex 50WX8 ion-exchange resin, and washed several times with water (100 mL) to remove excess salt. The amine product was then eluted with 28% aq $NH_3$ (60 mL) and the eluent was concentrated under reduced pressure. The off-white residue obtained was resuspended in 1 M HCl and concentrated to dryness to give the amine salt as a single isomer (67 mg, 40%); $^1$H NMR (600 MHz, $D_2O$) ŭ = 4.21 (dd, *J* = 5.0, 1.8, 1H, C*H*CHN), 4.01 (dd, *J* = 12.3, 4.5, 1H, CH*H*CHN), 3.86 (dd, *J* = 12.3, 3.0, 1H, CH*H*CHN), 3.84 (dd, *J* = 12.0, 1.8, 1H, CH*H*CHOH), 3.79 (ddd, *J* = 8.5, 6.0, 3.0, 1H, C*H*CH$_2$OH), 3.69 (dd, *J* = 12.0, 6.0, 1H, CH*H*CHOH), 3.68 (dd, *J* = 8.5, 1.8, 1H, C*H*CHCH$_2$OH), 3.64ï3.54 (m, 1H, C*H*N); $^{13}$C NMR (151 MHz, $D_2O$ with MeOH standard) ŭ = 71.0 (*C*HCH$_2$OH), 70.6 (*C*HCHCH$_2$OH), 66.9 (*C*HCHN), 63.0 (*C*H$_2$CHOH), 58.7 (*C*H$_2$CHN), 56.1 (*C*HN) ppm; HRMS (ES+) calcd for $C_6H_{16}NO_5$ $[M-Cl]^+$ 182.1028 found 182.1027. Characterisation data was in agreement with the literature.[14]

**Preparative scale biocatalytic reactions for D-fructose with IPA to give the 2-amino-2-deoxy-D-glucitol hydrochloride (*epi*-9)**

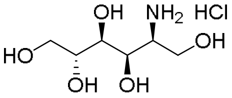


The enzymatic reaction was performed using a total volume of 30 mL, containing IPA (250 mM) as amine donor, and D-fructose (25 mM, 0.75 mmol), PLP (2 mM), potassium phosphate buffer pH 8.0 (50 mM), 25% v/v DMSO, clarified cell lysate of the enzyme cloned from pQR2191 (3.6 mL) at 45 °C and 250 rpm for 48 h. The reaction mixture was quenched with MeOH and centrifuged (10 min, 4000 rpm). The supernatant was basified to pH 12 with NaOH and concentrated to remove excess IPA. The yellow residue was redissolved in water (50 mL, pH adjusted to 8.0), loaded on to a Dowex 50WX8 ion-exchange resin, and washed several times with water (100 mL) to remove excess salt. The amine product was then eluted with 28% aq $NH_3$ (60 mL) and the eluent was concentrated under reduced pressure. The off-white residue obtained was resuspended in 1 M HCl and concentrated to dryness to give the amine salt as a single isomer (31.8 mg, 21%); $^{1}$H NMR (600 MHz, $D_2O$) ǔ = 4.10 (dd, *J* = 6.7, 1.3, 1H, C*H*CHN), 3.88 (dd, *J* = 12.0, 4.2, 1H, CH*H*CHN), 3.84 (dd, *J* = 12.0, 2.8, 1H, CH*H*CHOH), 3.77 (dd, *J* = 12.0, 6.6, 1H, CH*H*CHN), 3.76 (dd, *J* = 9.0, 1.2, 1H, C*H*$CH_2$OH), 3.67 (dd, *J* = 12.0, 6.0, 1H, CH*H*CHOH), 3.64 (dd, *J* = 8.5, 1.8, 1H, C*H*CH$CH_2$OH), 3.56ï 3.50 (m, 1H, C*H*N); $^{13}$C NMR (151 MHz, $D_2O$ with MeOH standard) ǔ = 70.9 (*C*H$CH_2$OH), 70.6 (*C*HCH$CH_2$OH), 66.1 (*C*HCHN), 62.9 (*C*$H_2$CHOH), 59.0 (*C*$H_2$CHN), 55.5 (CHN) ppm; HRMS (ES+) calcd for $C_6H_{16}NO_5$ $[M-Cl]^+$ 182.1028 found 182.1027.

## V. NMR spectra

**(2*S*,3*S*,4*S*)-2-(Dihydroxymethyl)tetrahydrofuran-3,4-diol (*anti*-2a) and (2*R*,3*S*,4*S*)-2-(dihydroxymethyl)tetrahydrofuran-3,4-diol (*syn*-2a)**

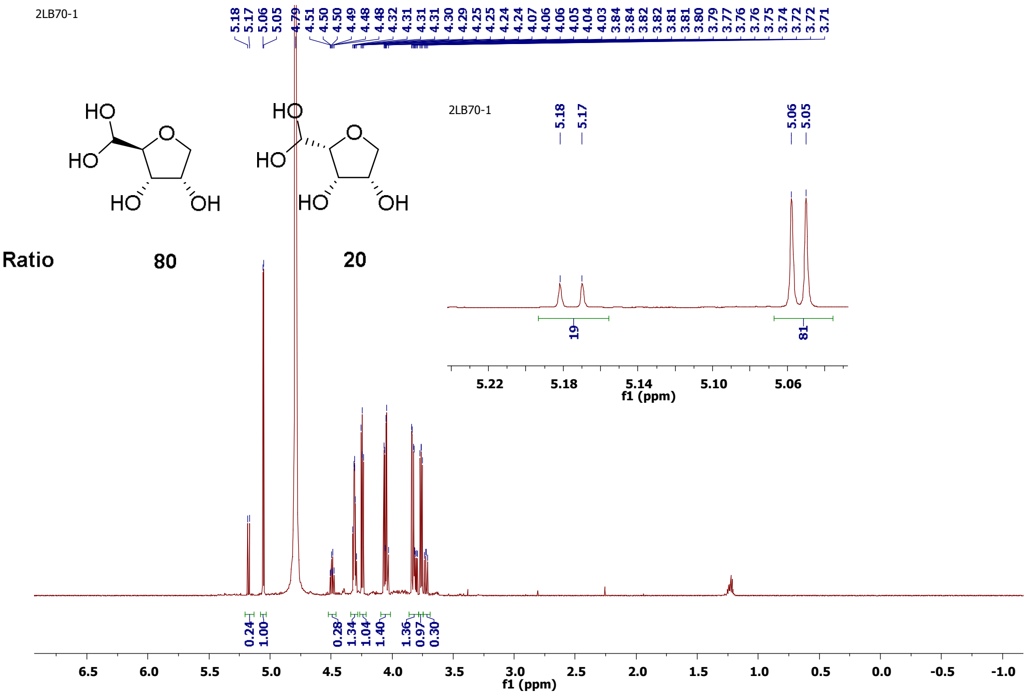


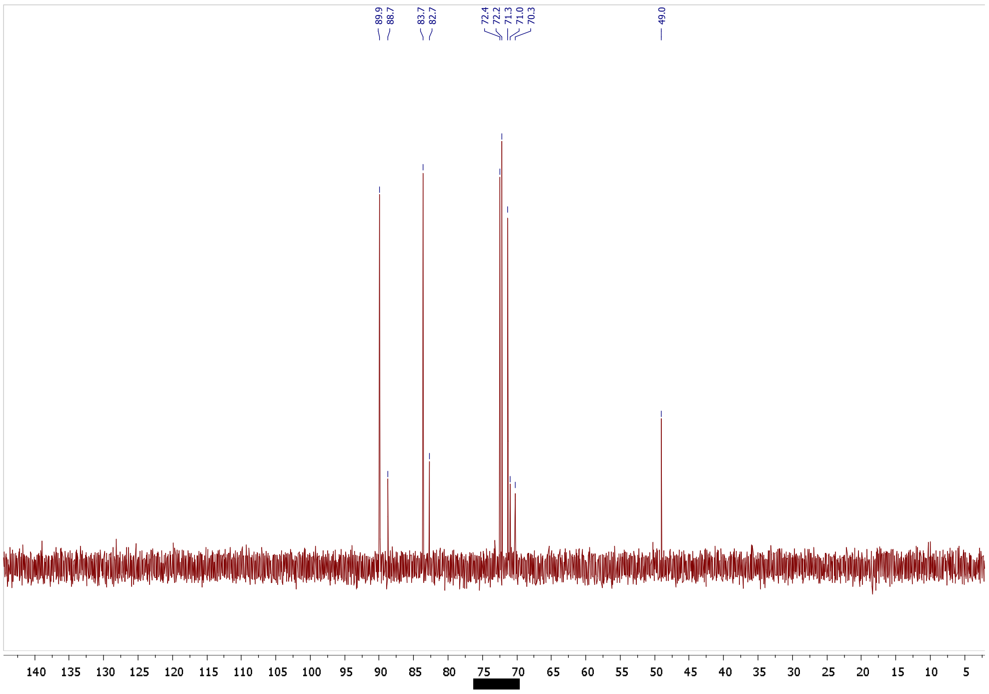

**(2*R*,3*R*,4*R*)-2-(Dihydroxymethyl)tetrahydrofuran-3,4-diol (*anti*-2b) and (2*S*,3*R*,4*R*)-2-(dihydroxymethyl)tetrahydrofuran-3,4-diol (*syn*-2b)**

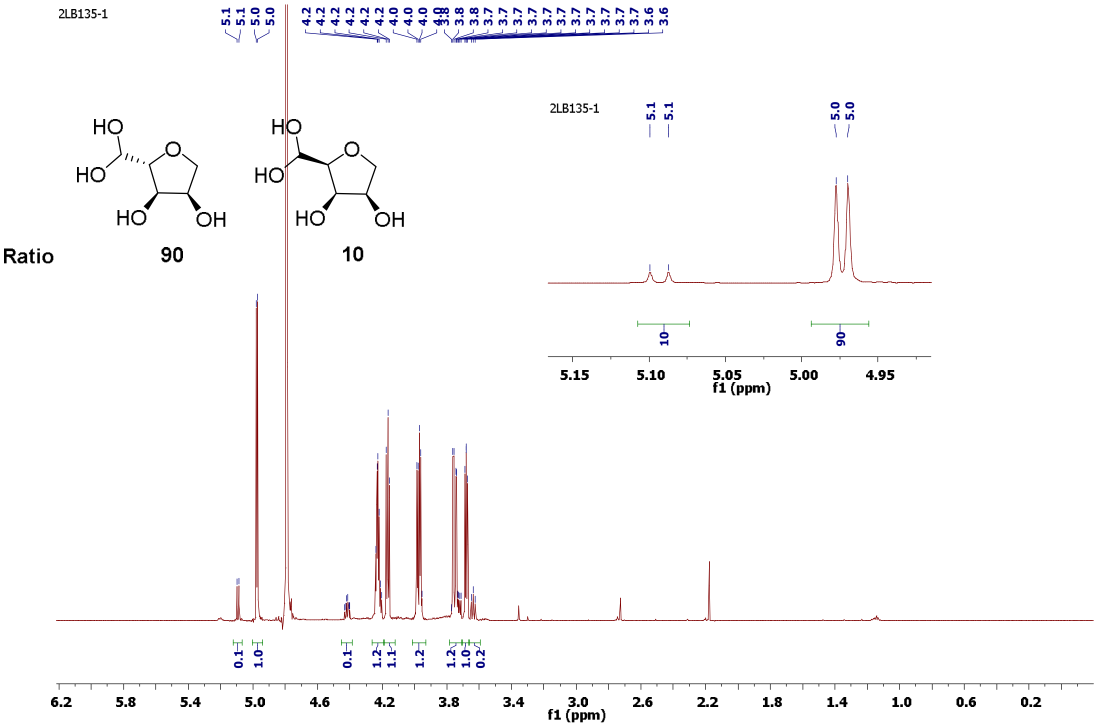


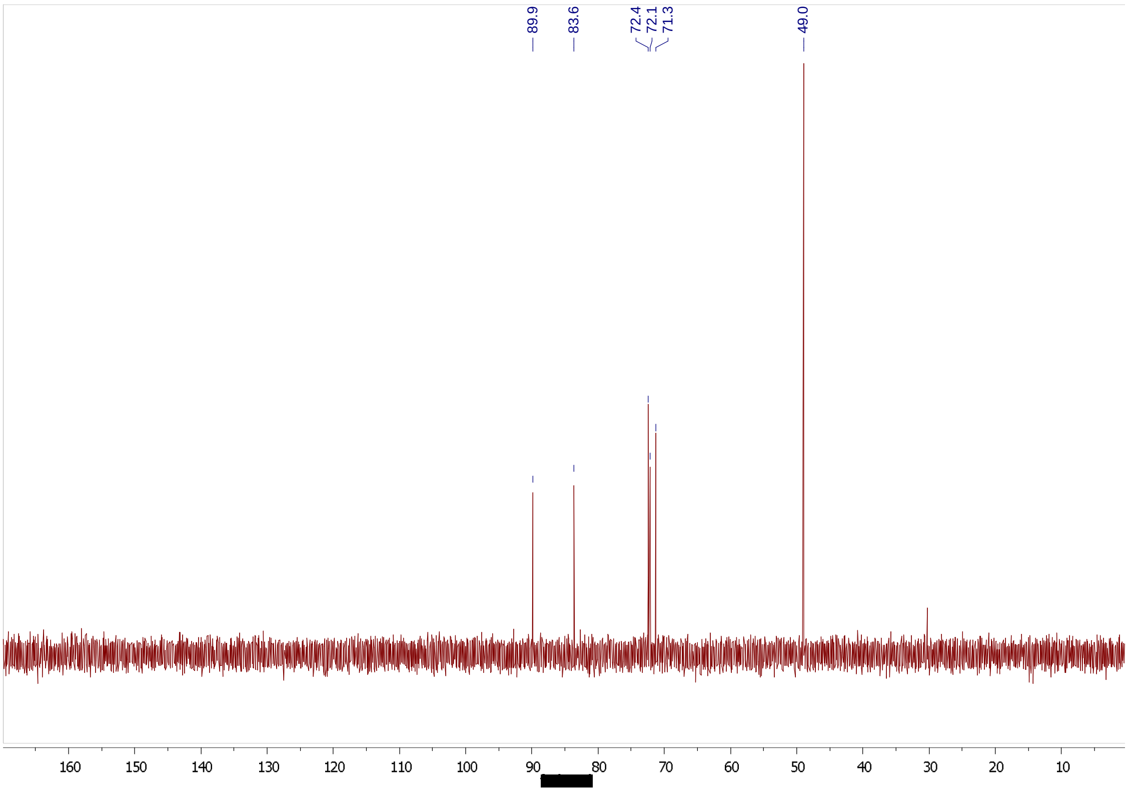

## (2*S*,3*S*,4*R*)-2-(Dihydroxymethyl)tetrahydrofuran-3,4-diol (*anti*-2c) and (2*R*,3*S*,4*R*)-2-(dihydroxymethyl)tetrahydrofuran-3,4-diol (*syn*-2c)

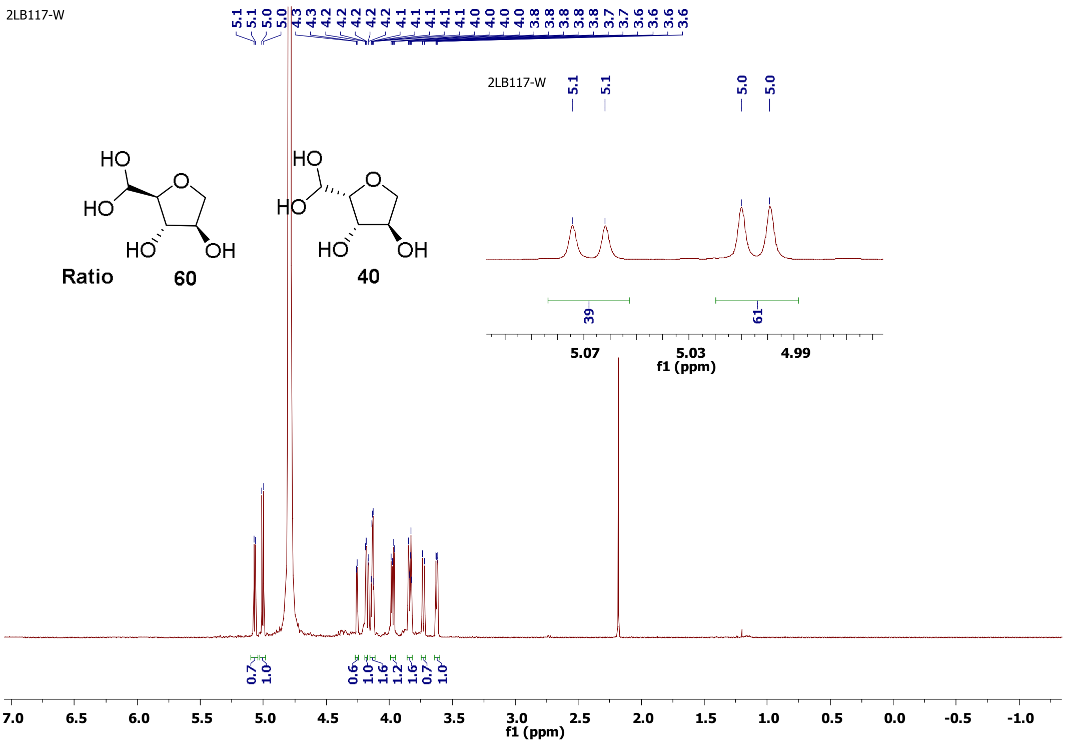


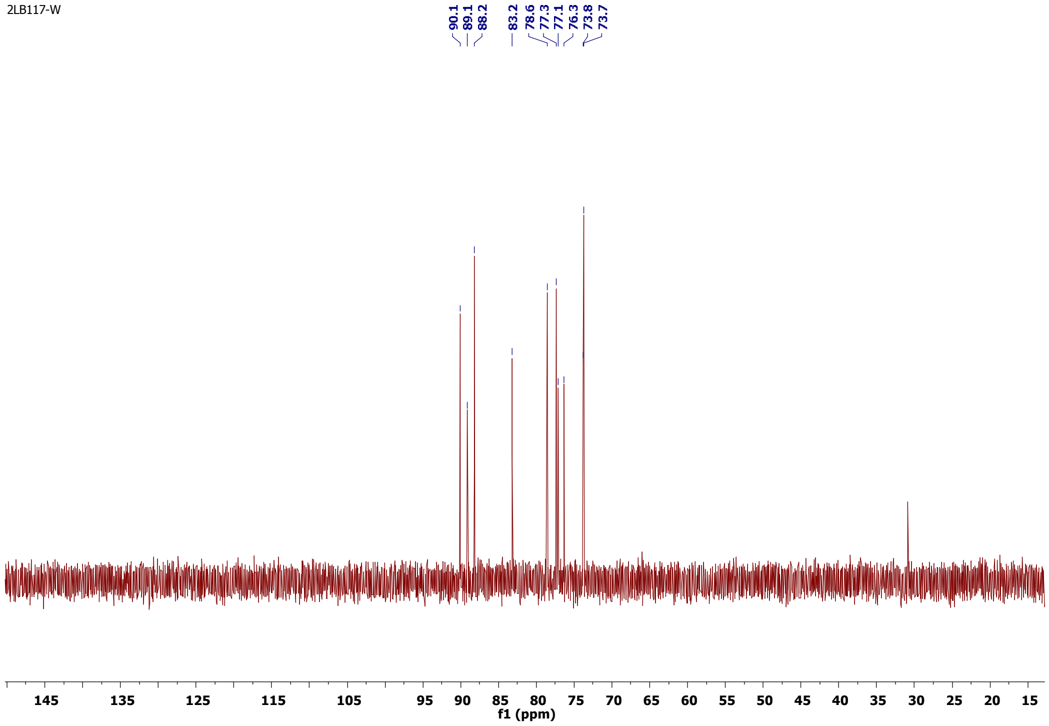

**(2*R*,3*R*,4*R*,5*S*)-2-(Dihydroxymethyl)-5-methyltetrahydrofuran-3,4-diol (*anti*-2d) and (2*S*,3*R*,4*R*,5*S*)-2-(dihydroxymethyl)-5-methyltetrahydrofuran-3,4-diol (*syn*-2d)**

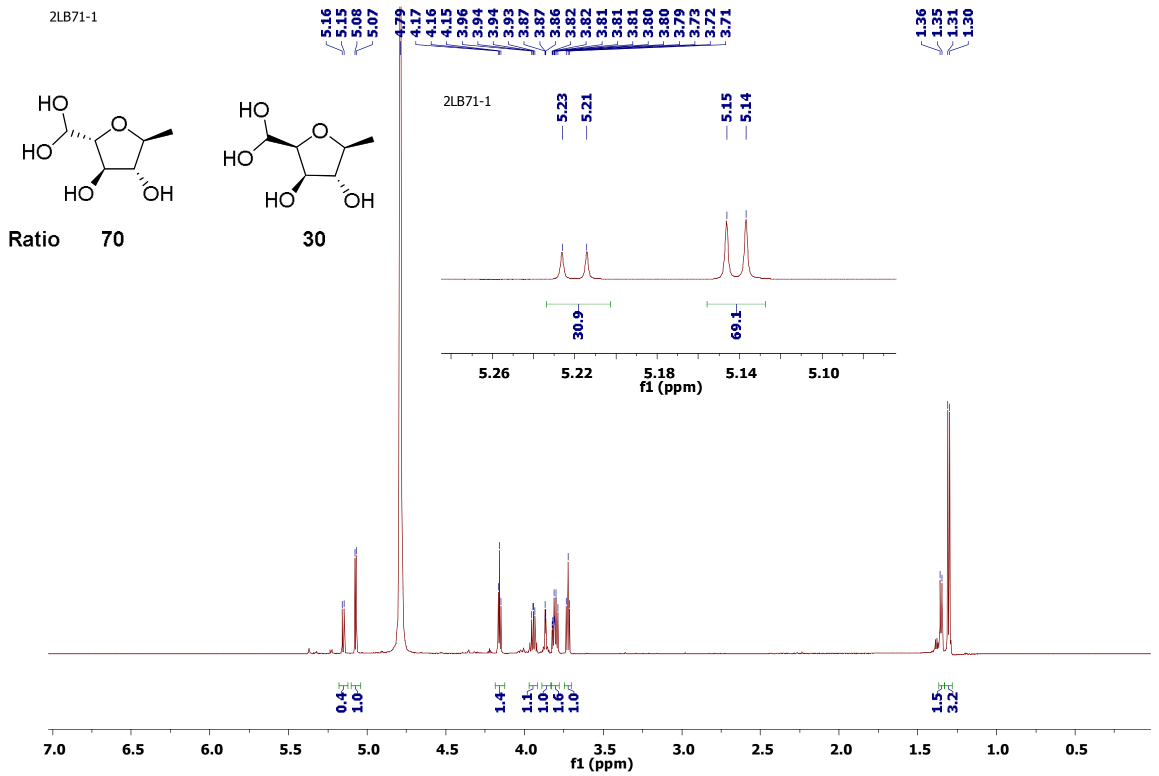


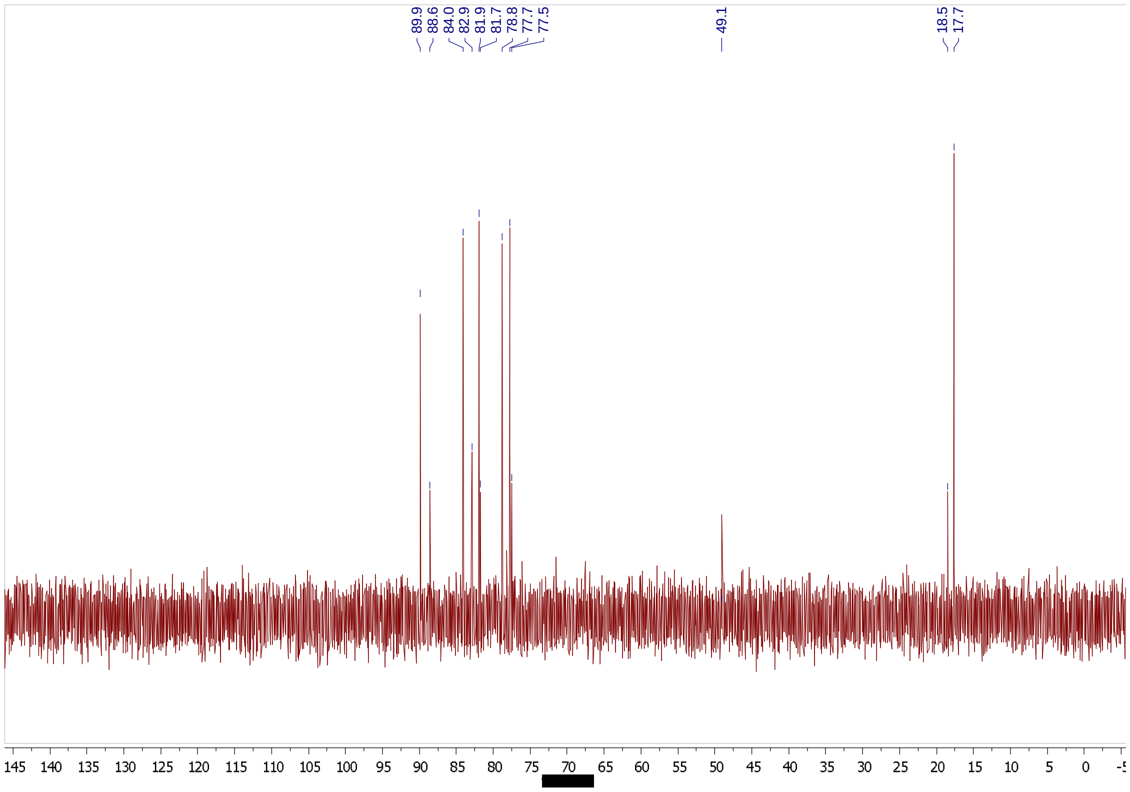

***tert*-Butyl (((2*R*,3*S*,4*S*)-3,4-dihydroxytetrahydrofuran-2-yl)methyl)carbamate (4a)**

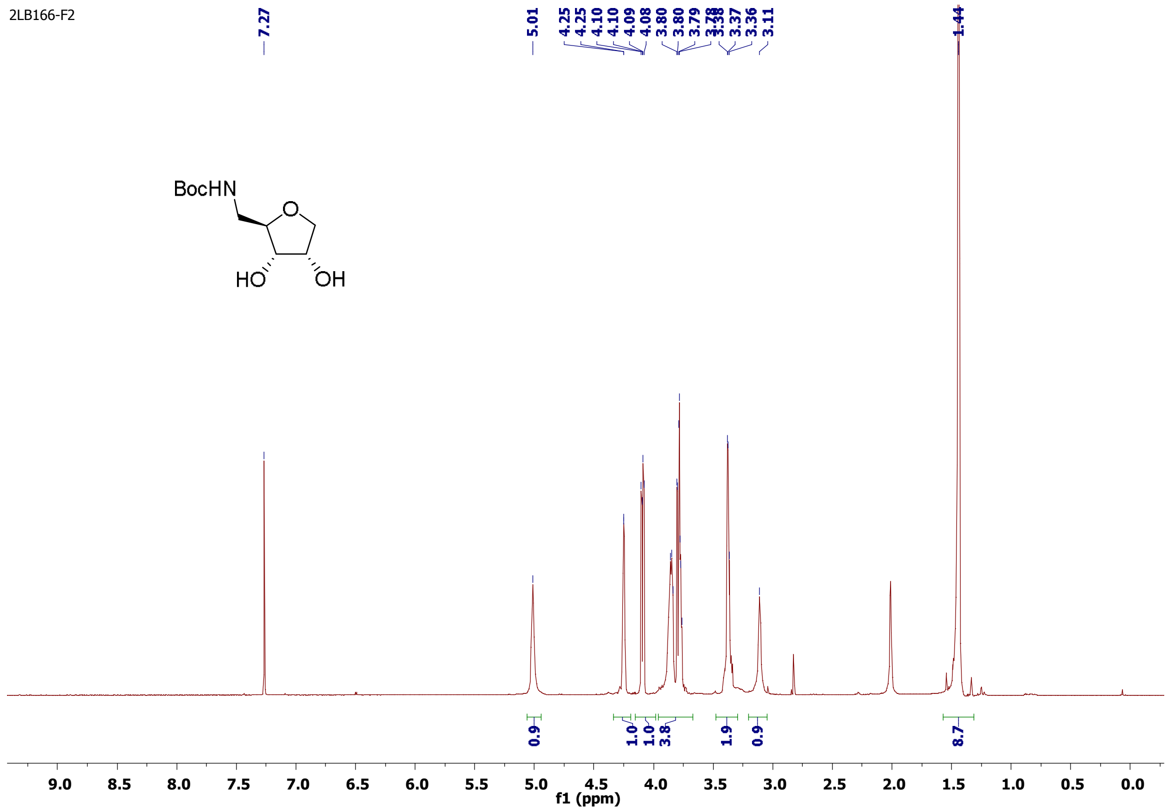


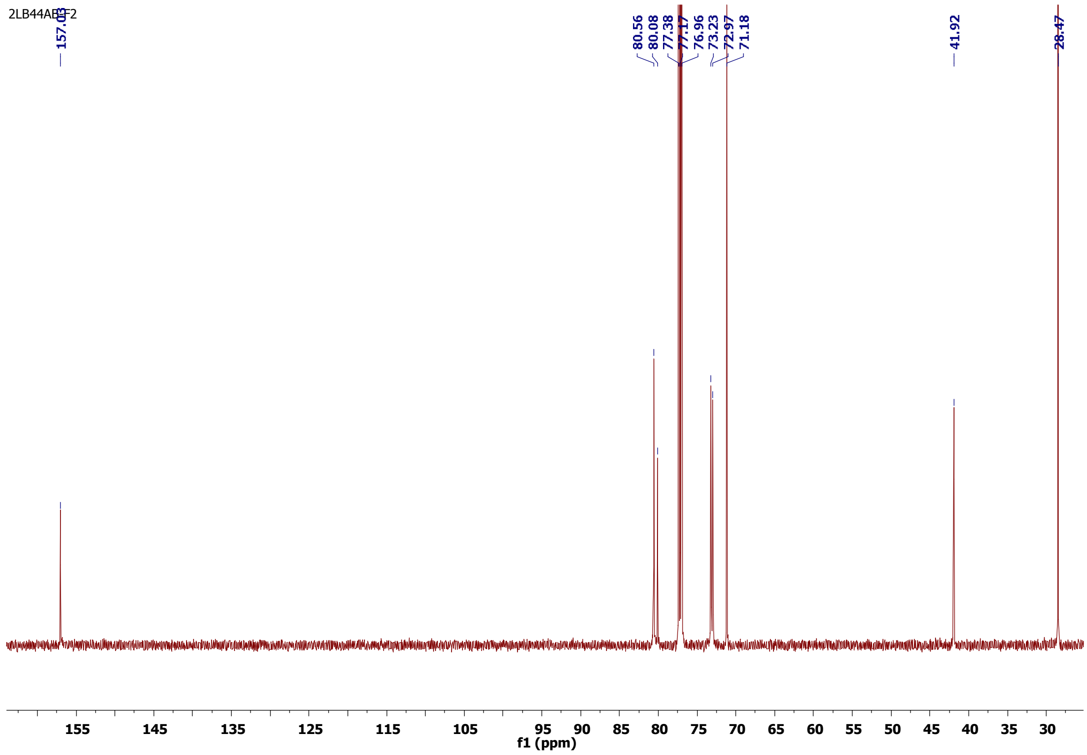

**_tert_-Butyl (((2_S_,3_R_,4_R_)-3,4-dihydroxytetrahydrofuran-2-yl)methyl)carbamate (4b)**

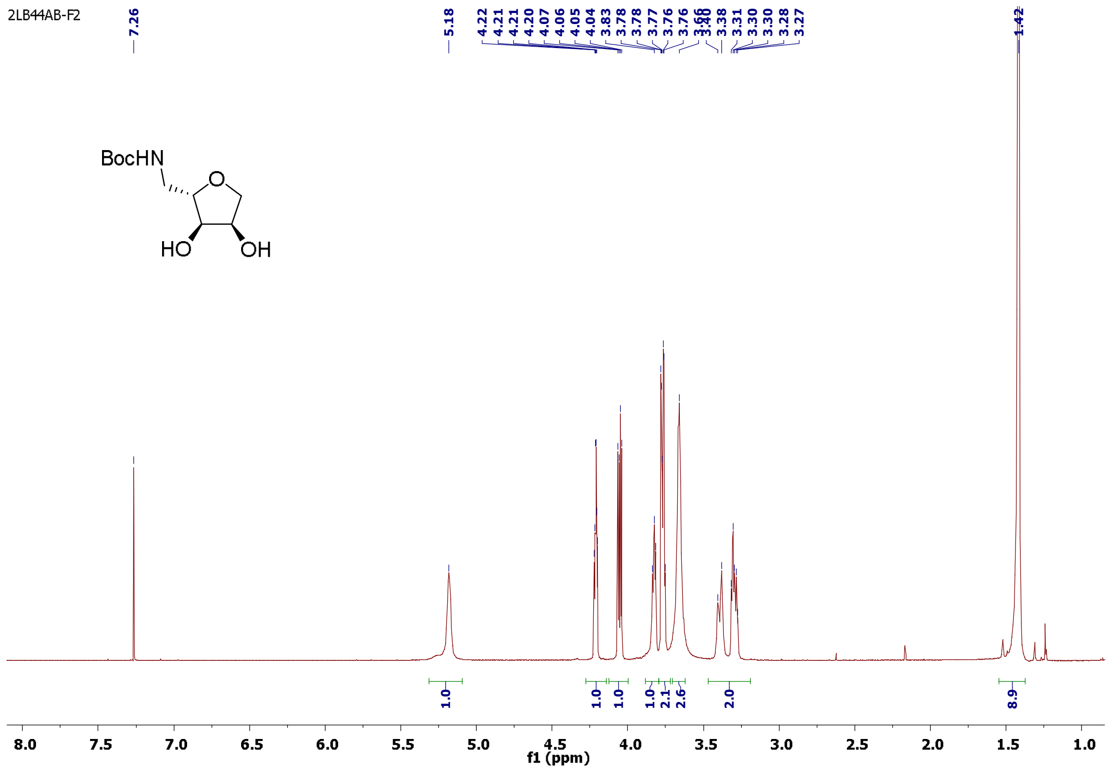


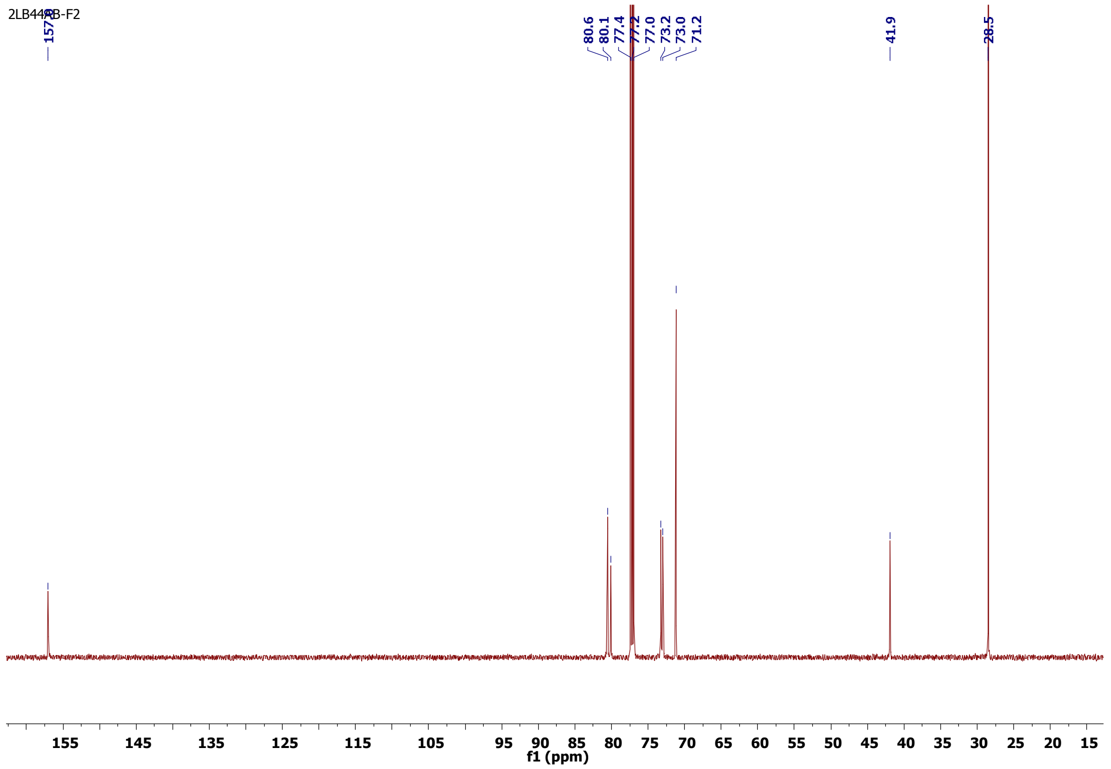

***tert*-Butyl (((2*R*,3*S*,4*R*)-3,4-dihydroxytetrahydrofuran-2-yl)methyl)carbamate (*anti*-4c) and *tert*-butyl (((2*S*,3*S*,4*R*)-3,4-dihydroxytetrahydrofuran-2-yl)methyl)carbamate (*syn*-4c)**

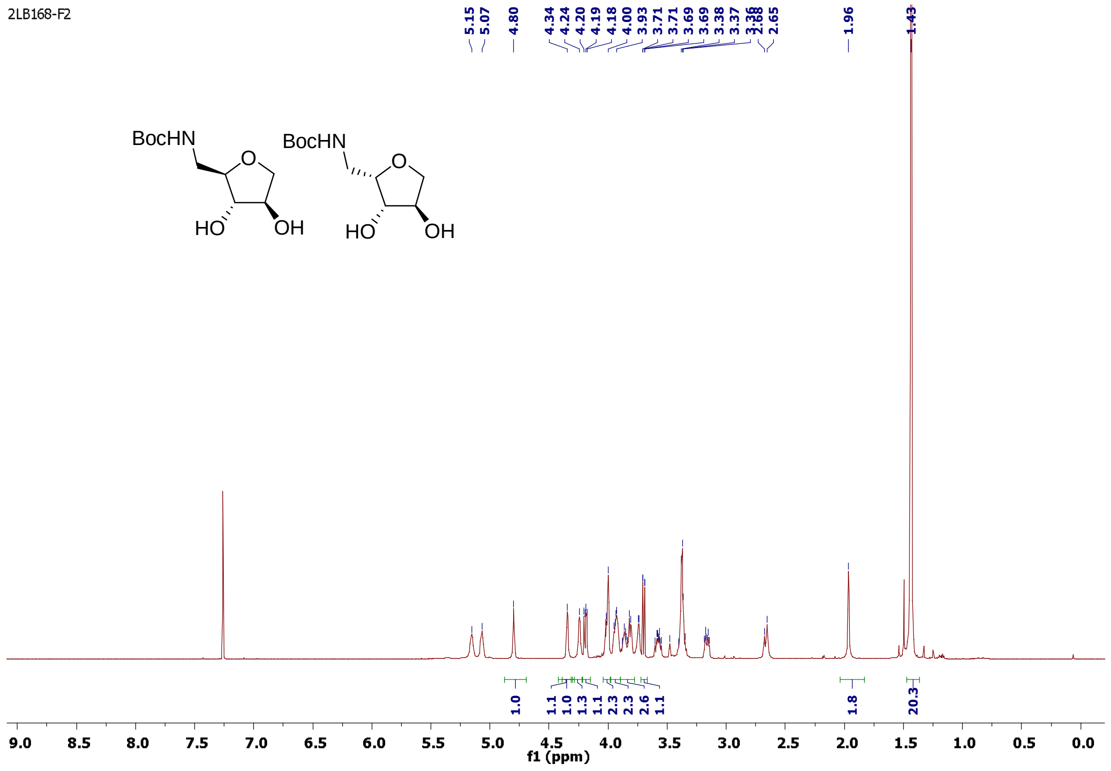


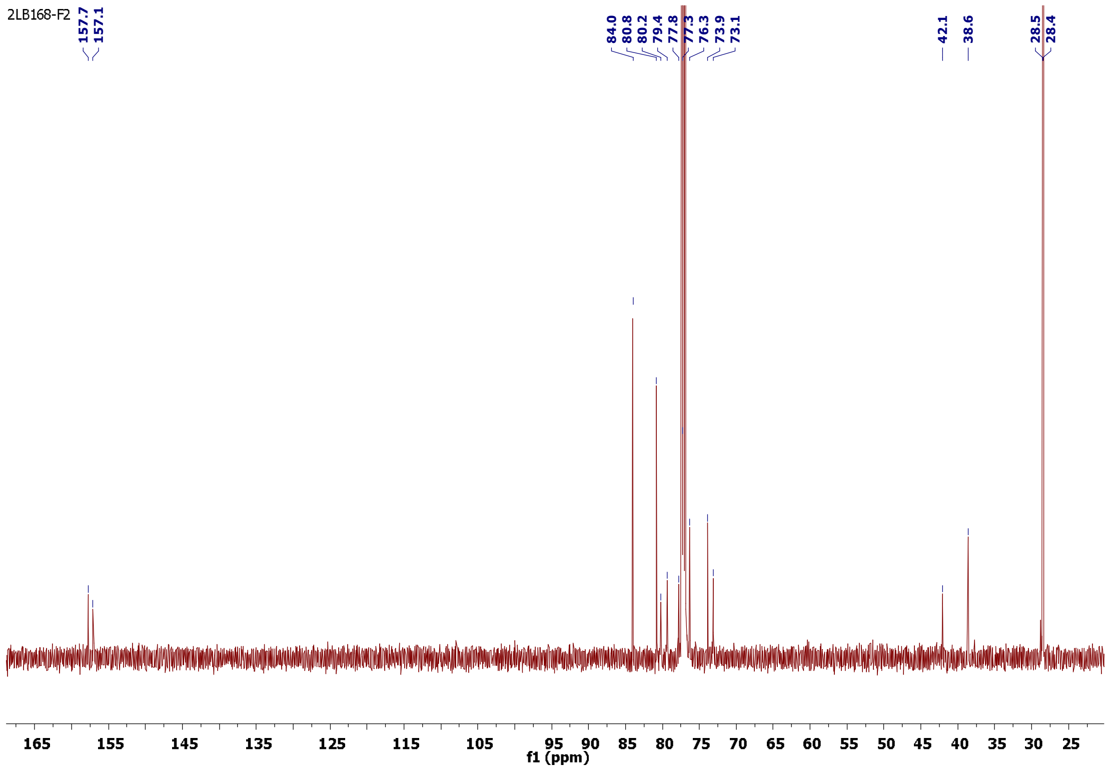

**tert-Butyl-(((2R,3R,4R,5S)-3,4-dihydroxy-5-methyltetrahydrofuran-2-yl)methyl)carbamate (anti-4d) and tert-butyl-(((2S,3R,4R,5S)-3,4-dihydroxy-5-methyltetrahydrofuran-2-yl)methyl)carbamate (syn-4d)**

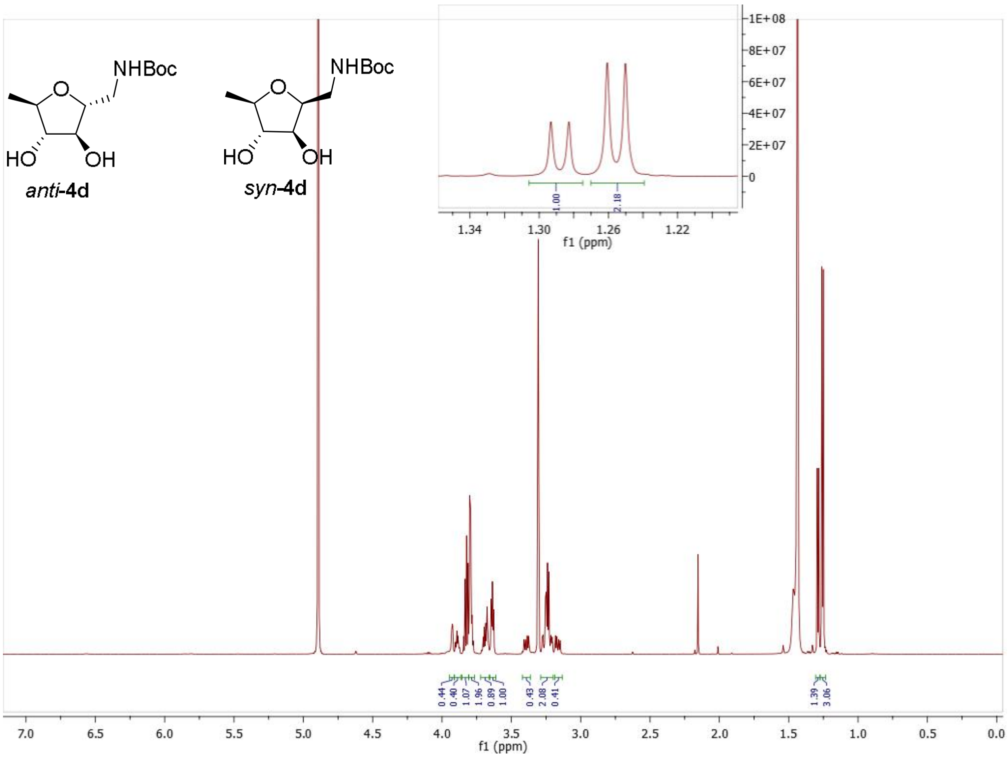


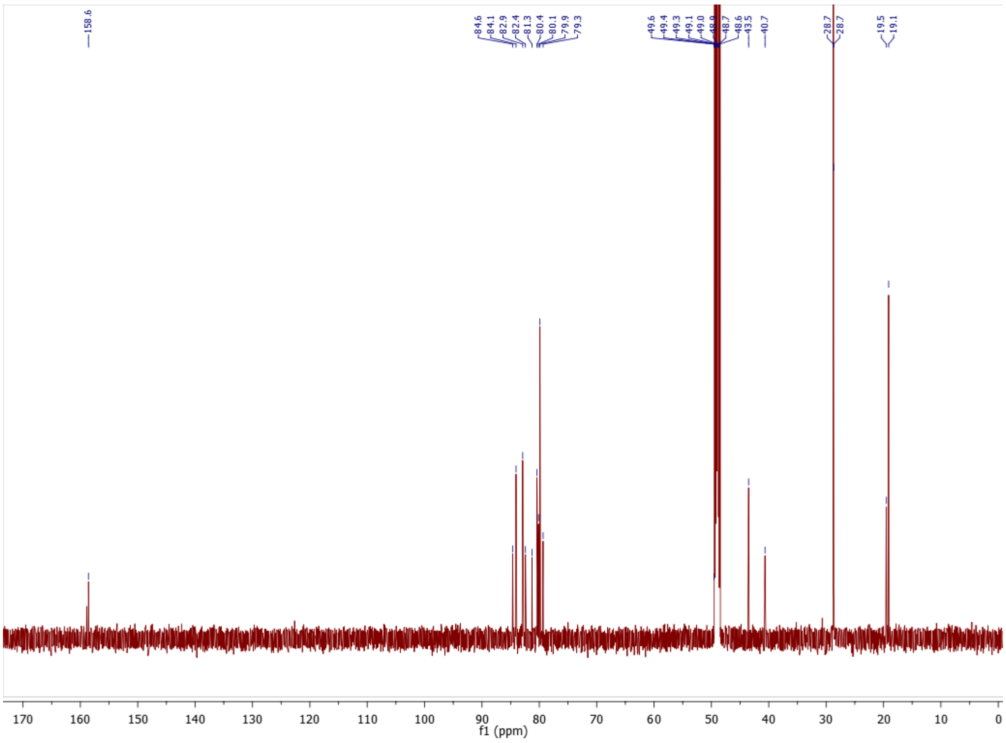

**(2*R*,3*S*,4*S*)-2-(Aminomethyl)tetrahydrofuran-3,4-diol hydrochloride (3a)**

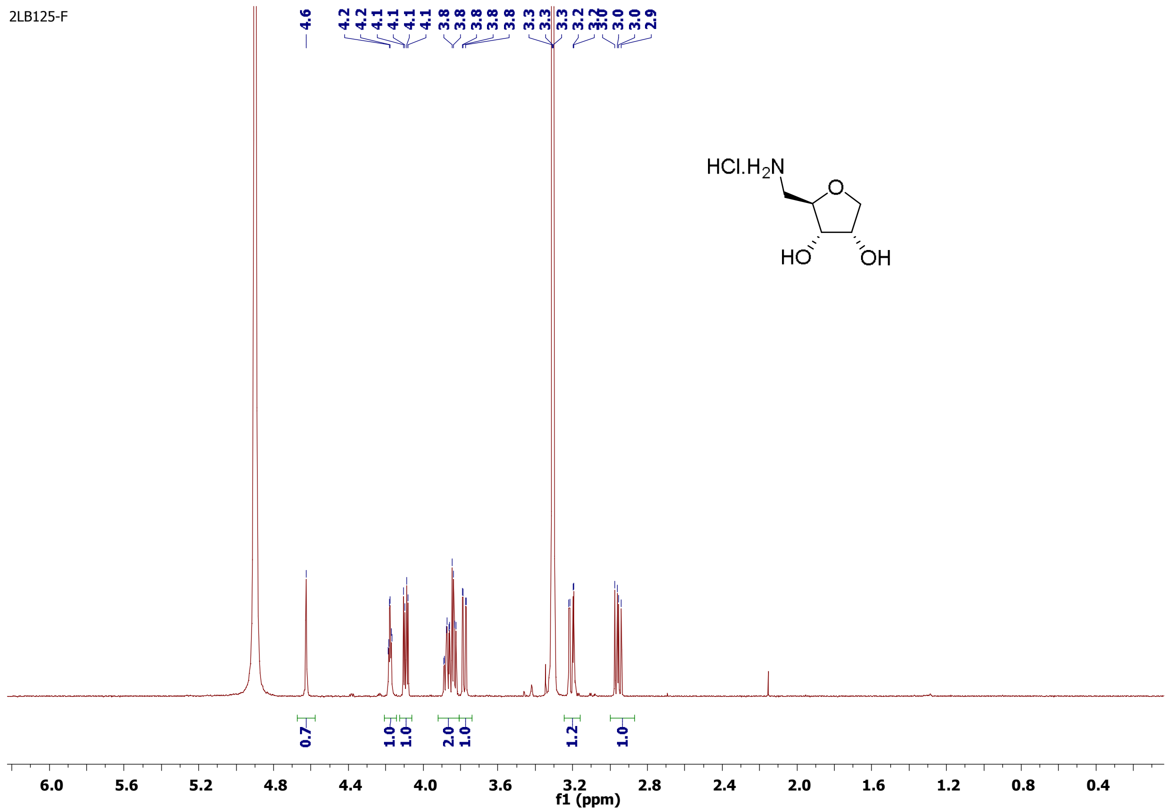


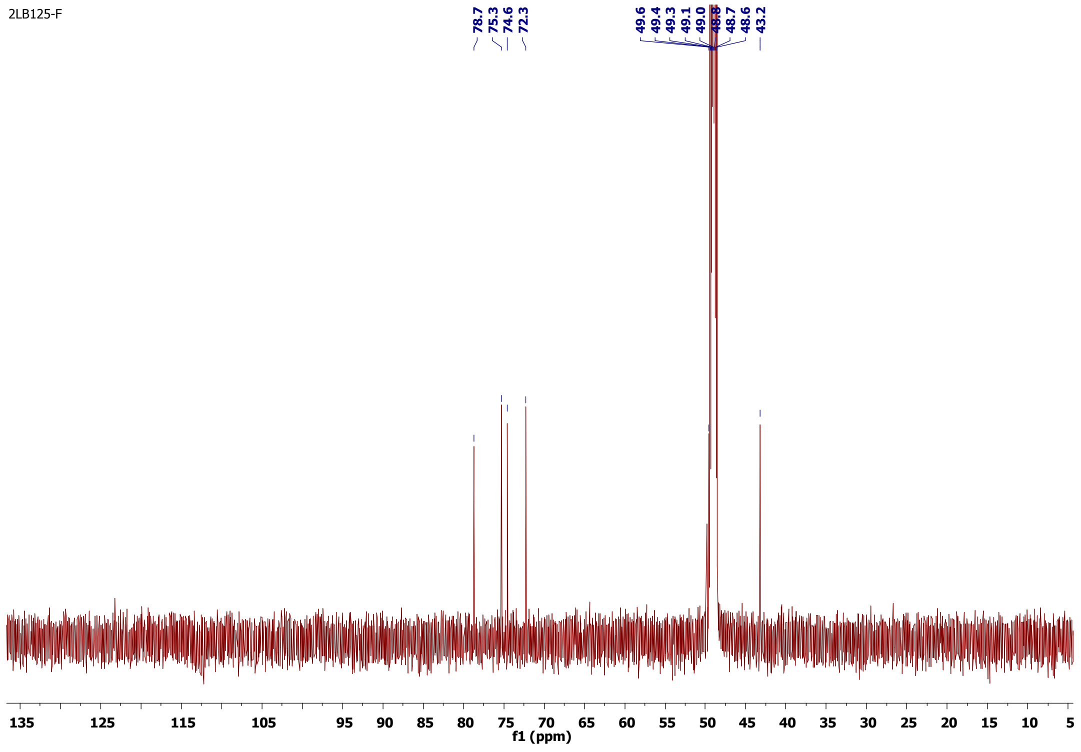

## (2*S*,3*R*,4*R*)-2-(Aminomethyl)tetrahydrofuran-3,4-diol hydrochloride (3b)

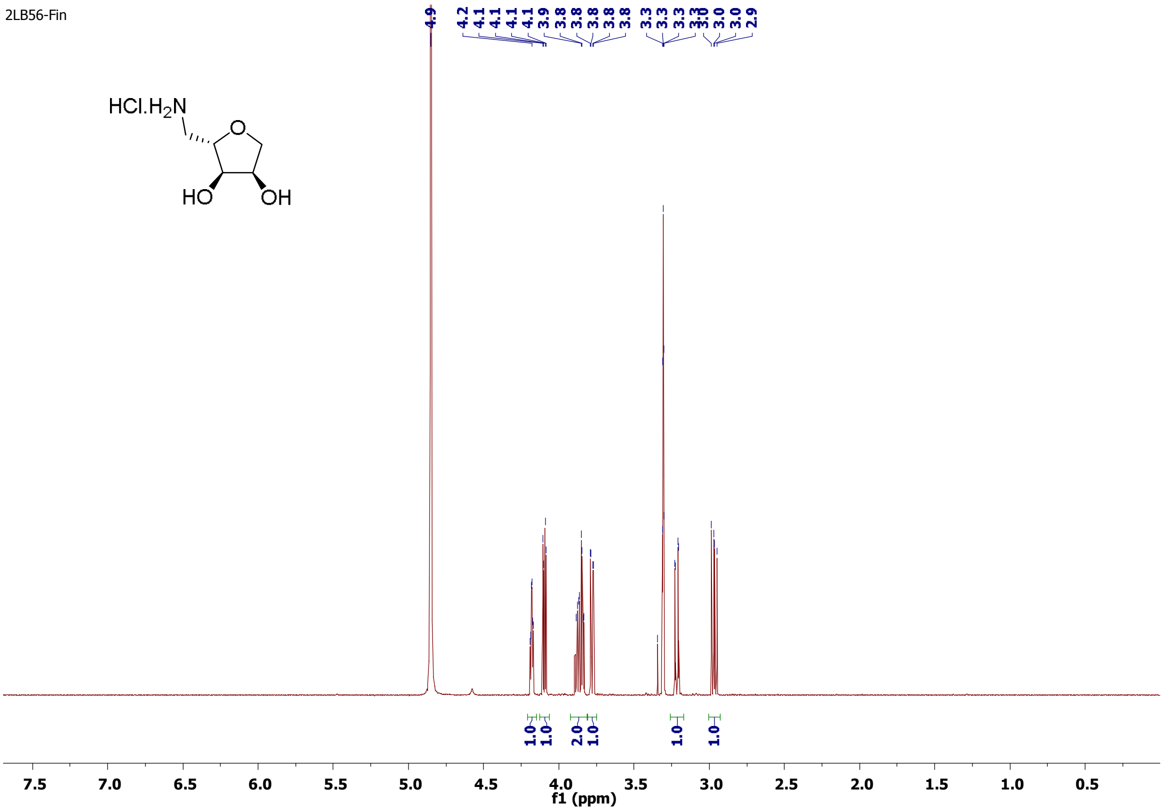


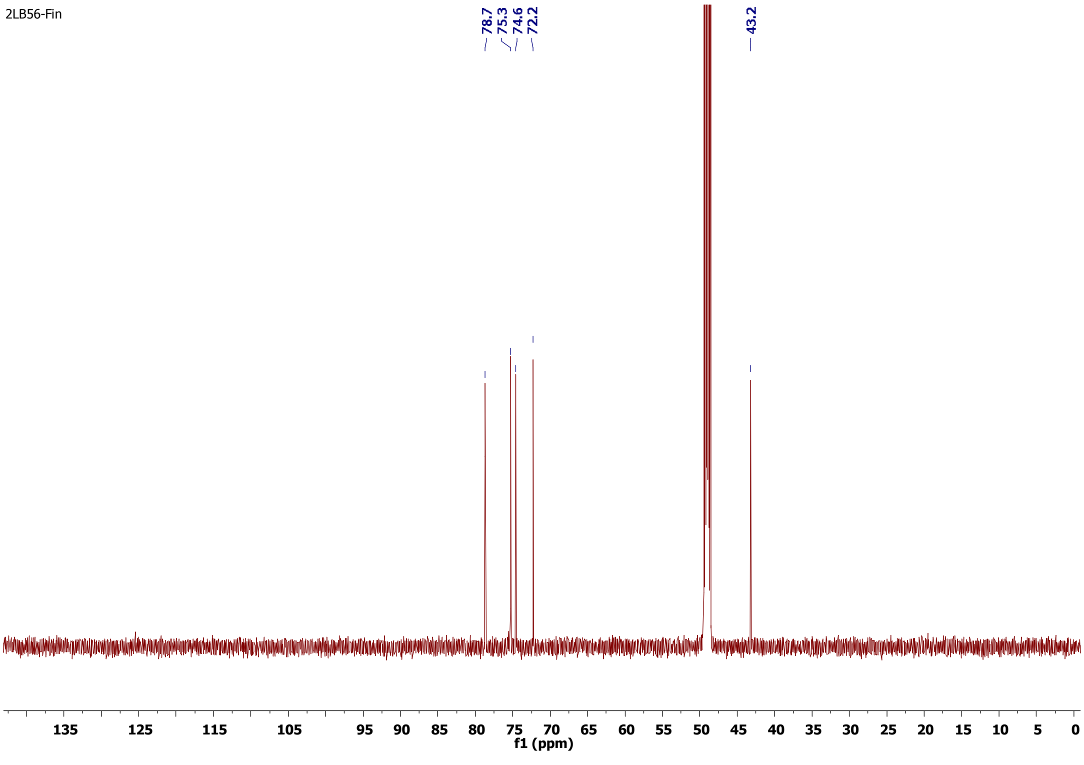

**(2*R*,3*S*,4*R*)-2-(Aminomethyl)tetrahydrofuran-3,4-diol hydrochloride (*anti*-3c) and (2*S*,3*S*,4*R*)-2-(aminomethyl)tetrahydrofuran-3,4-diol hydrochloride (*syn*-3c)**

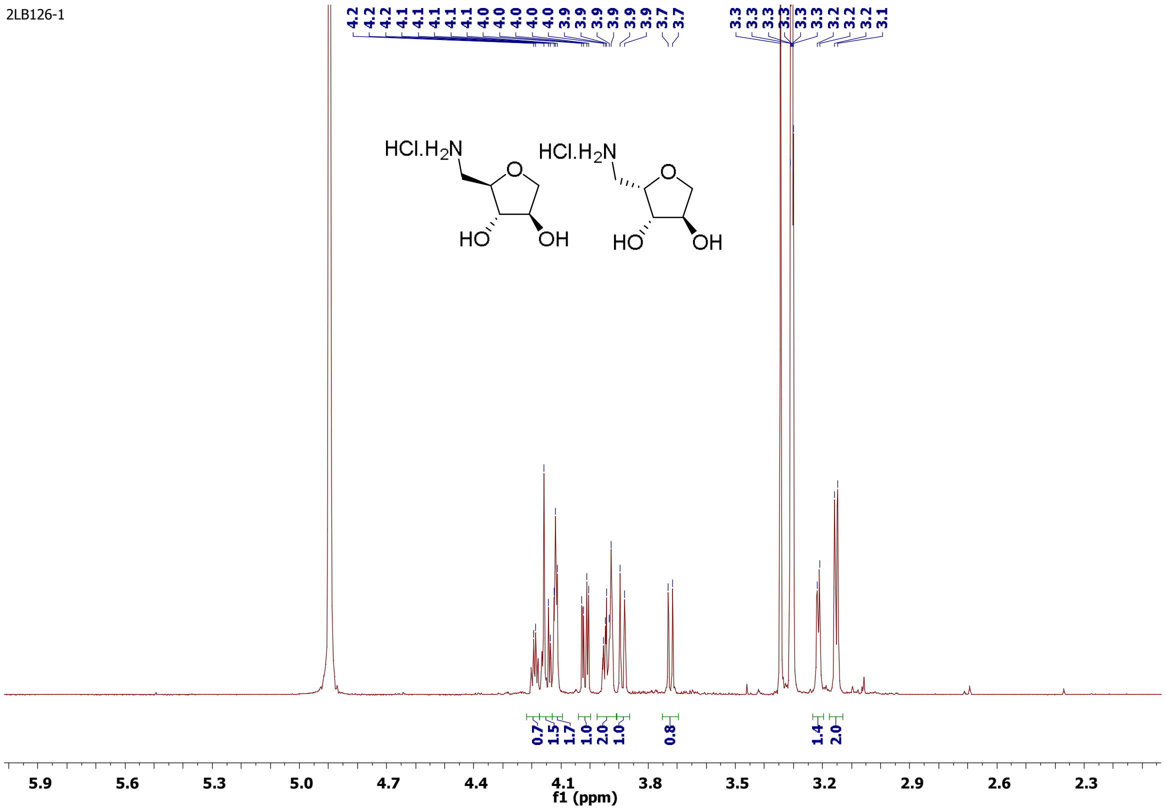


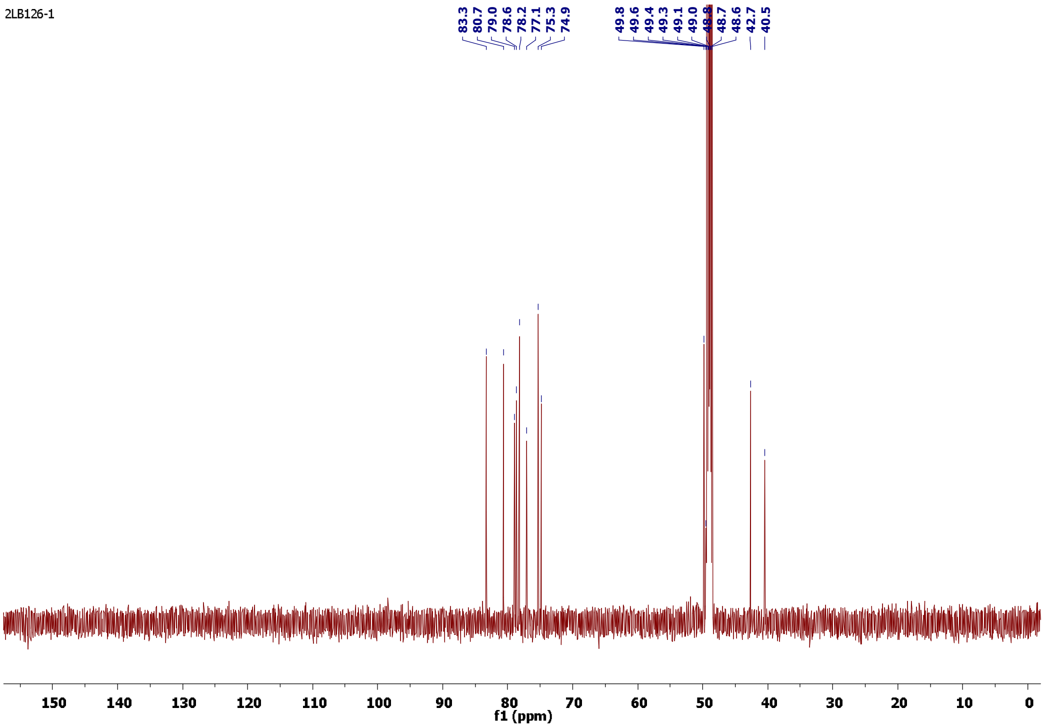

**(2*R*,3*R*,4*R*,5*S*)-2-(Aminomethyl)-5-methyltetrahydrofuran-3,4-diol hydrochloride (*anti*-3d) and (2*S*,3*R*,4*R*,5*S*)-2-(aminomethyl)-5-methyltetrahydrofuran-3,4-diol hydrochloride (*syn*-3d)**

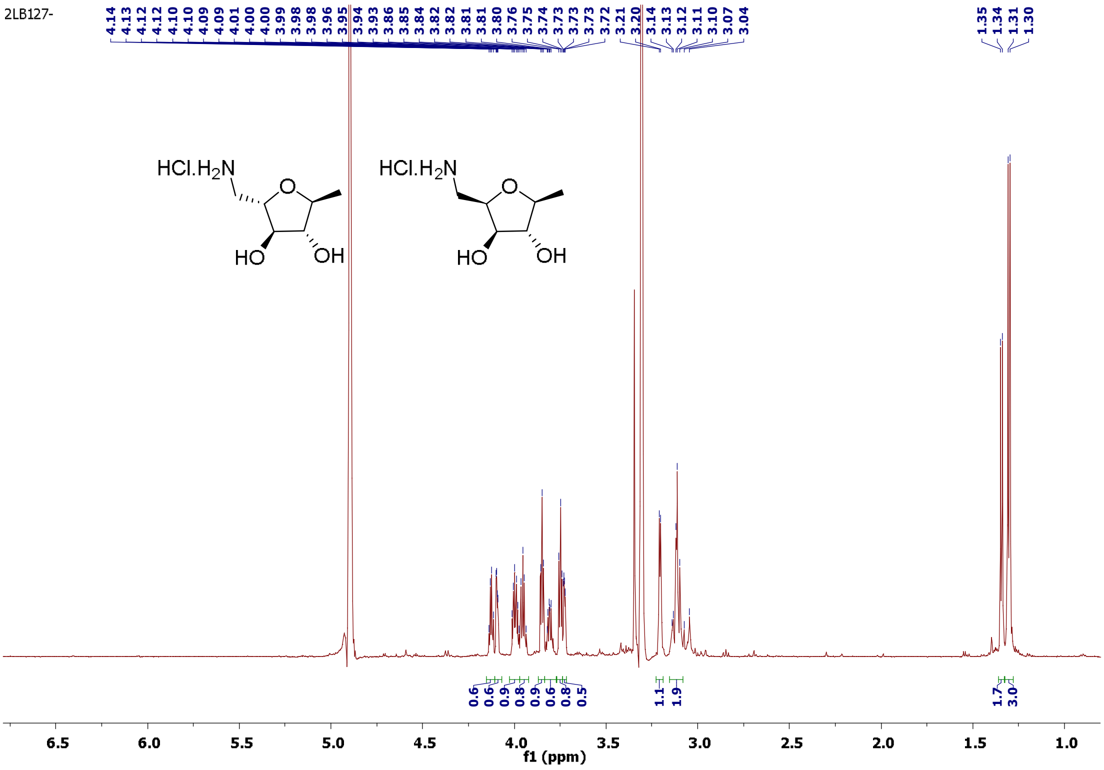


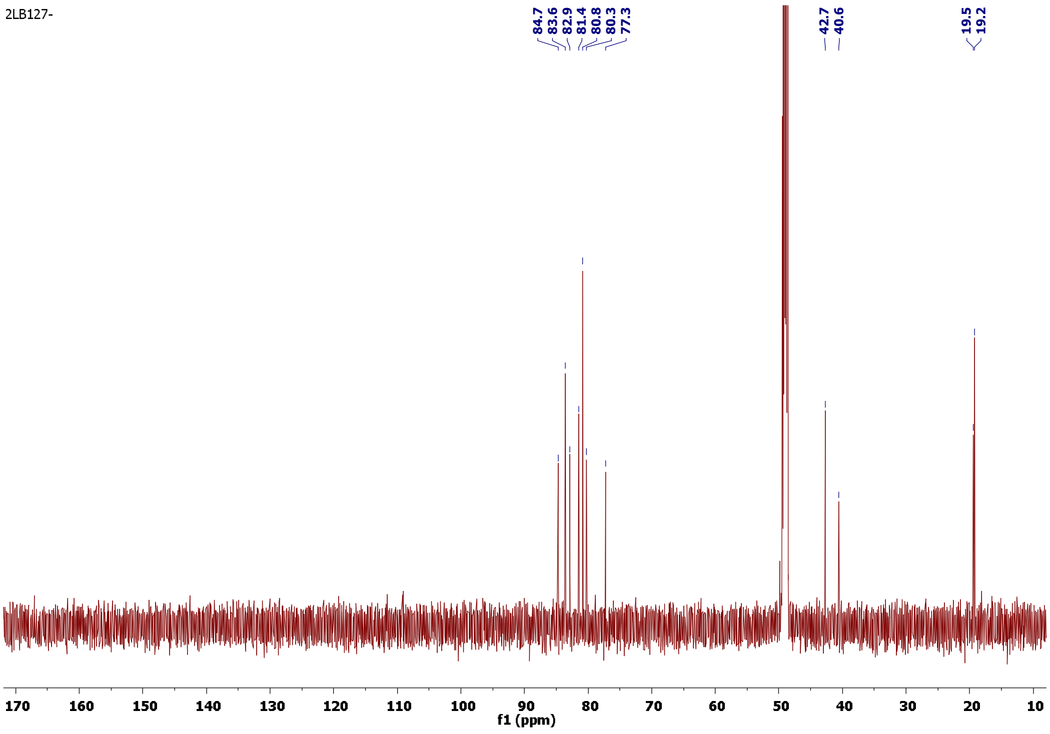

**1-Amino-1-deoxy-L-arabinitol hydrochloride[13] (7)**

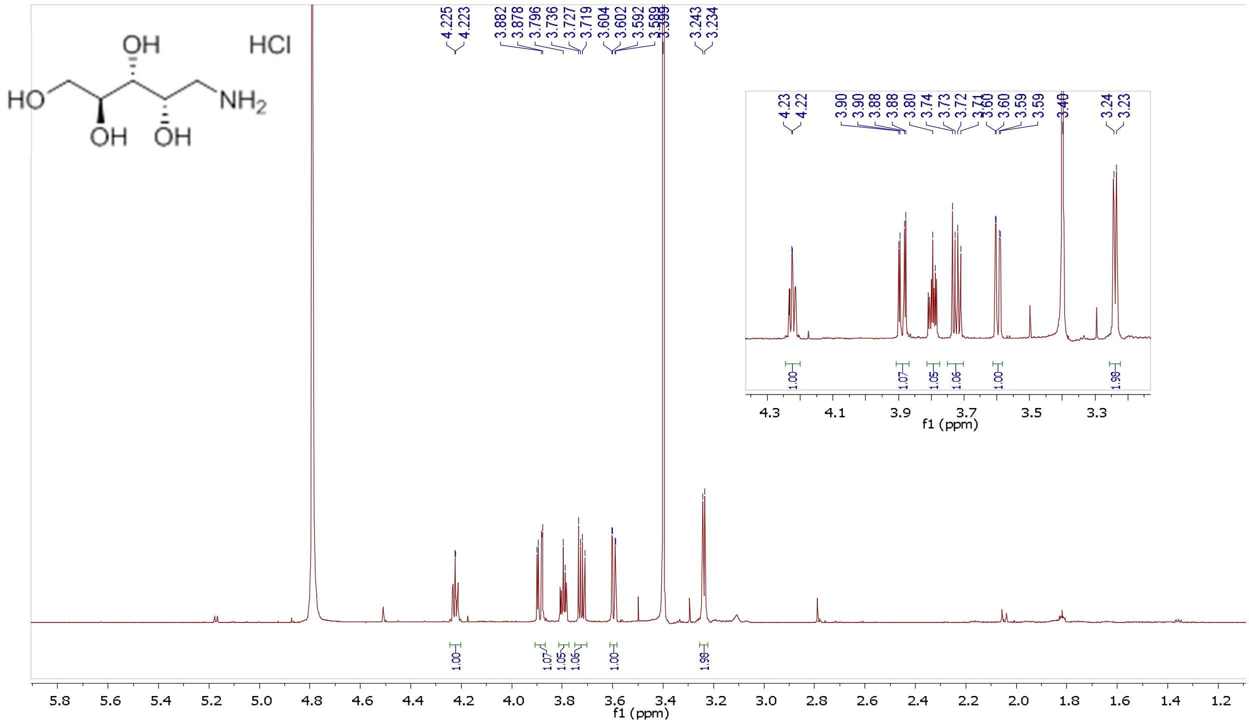


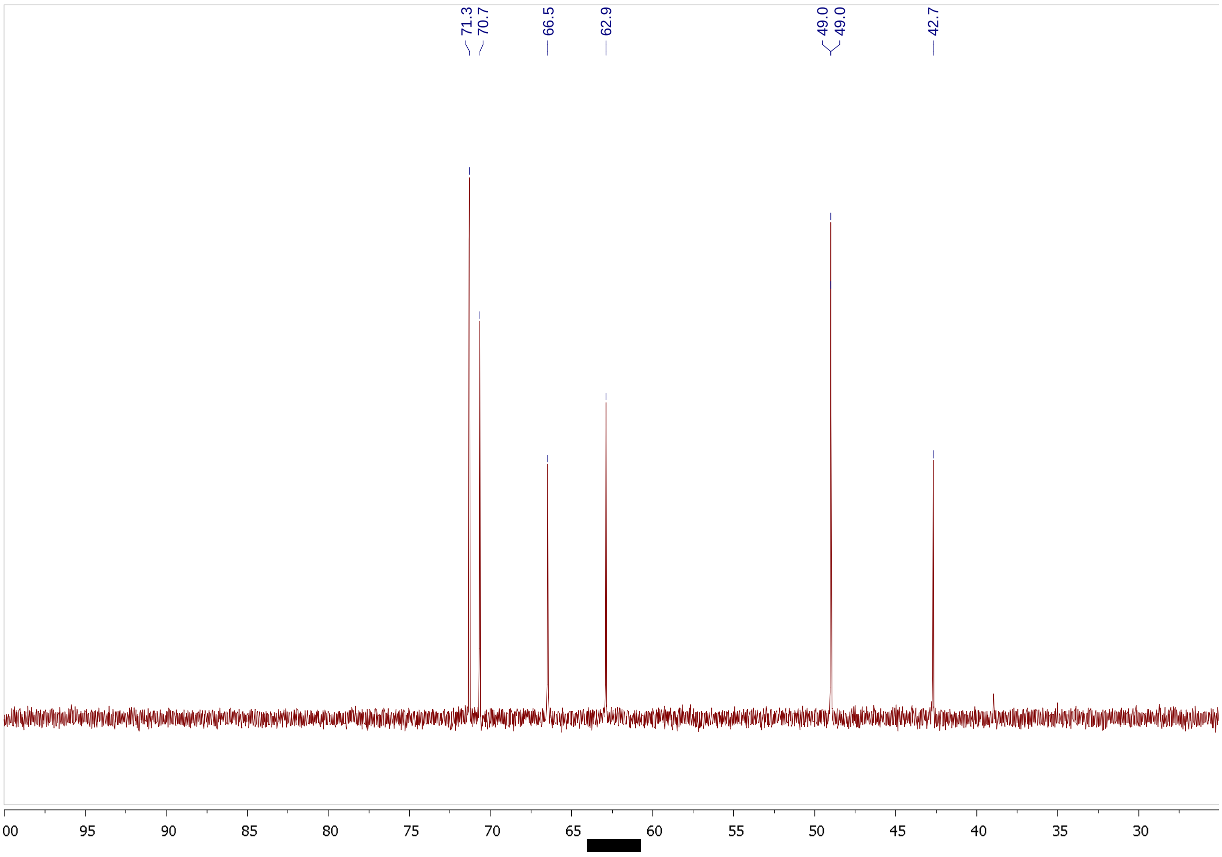

## 1-Amino-1-deoxy-D-xylitol hydrochloride (8)

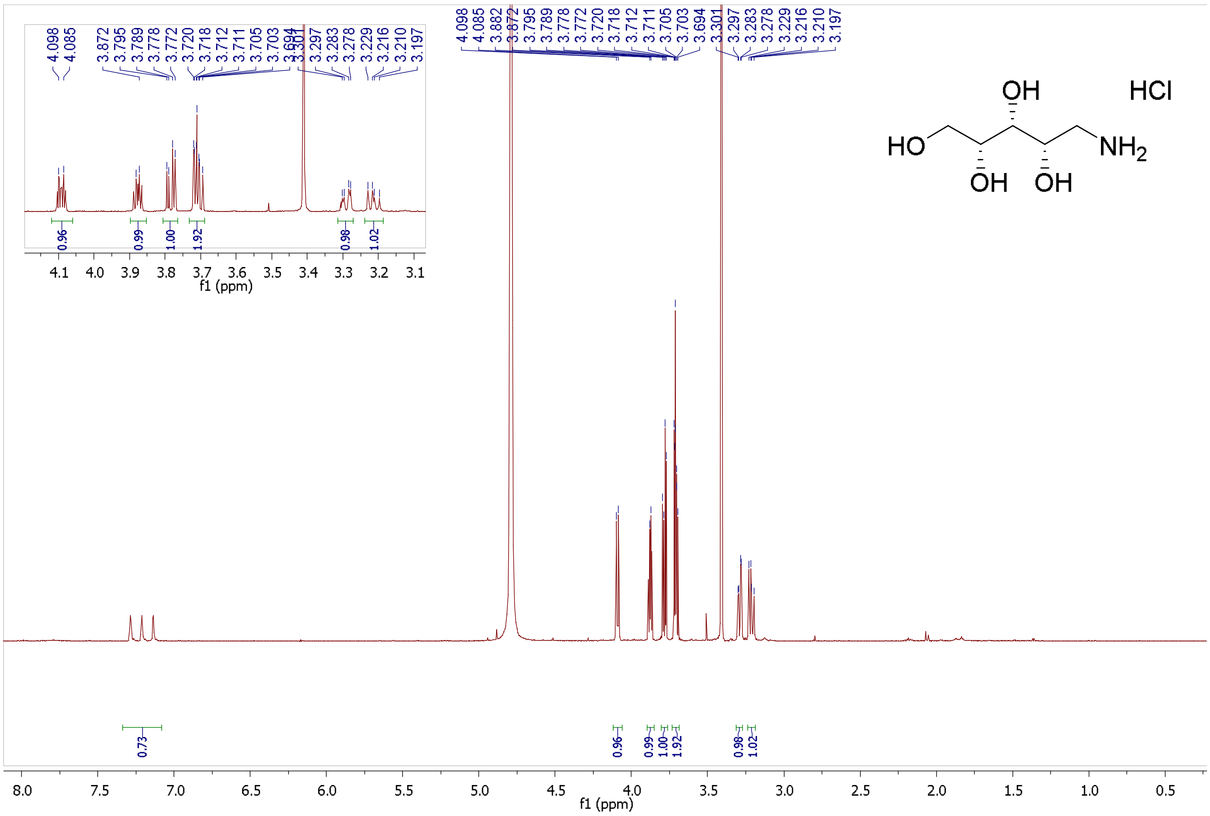


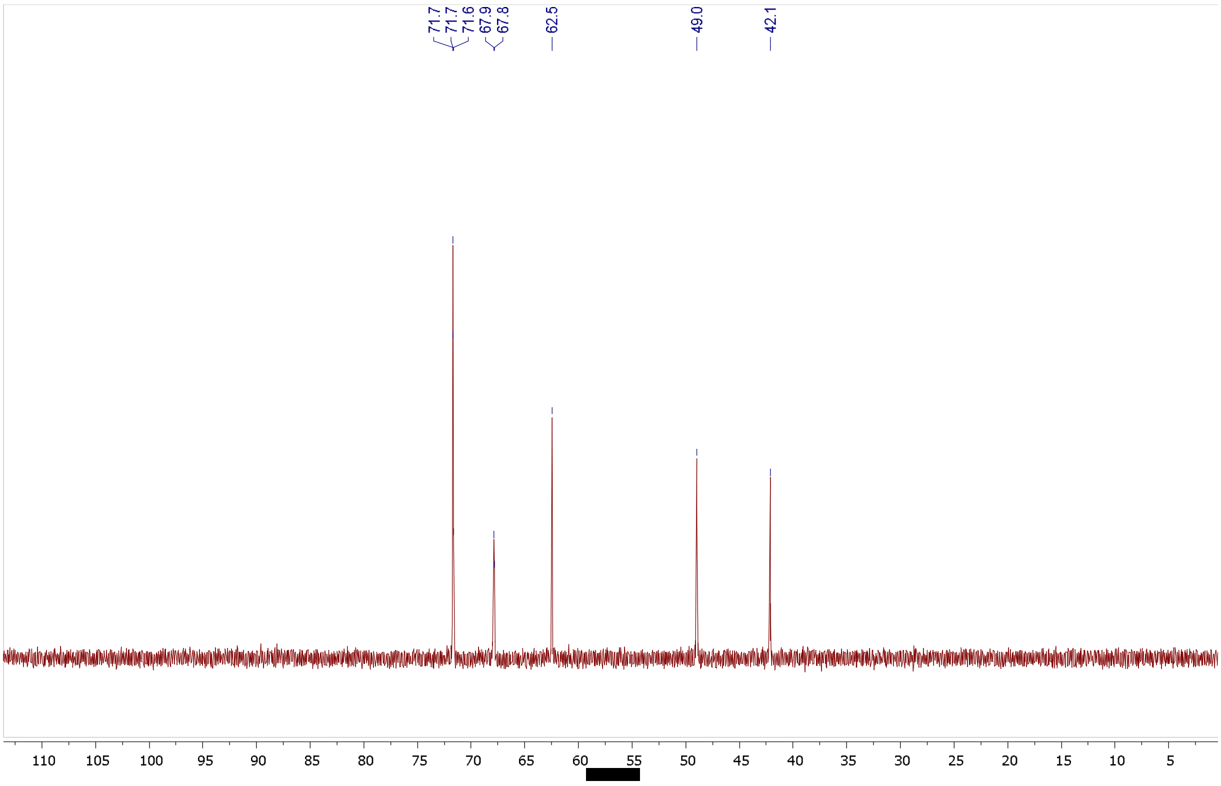

**1-Amino-1-deoxy-D-xylitol hydrochloride (8)**

with $NH_4Cl$ removed ($^{1}H$ NMR, 400 MHz)

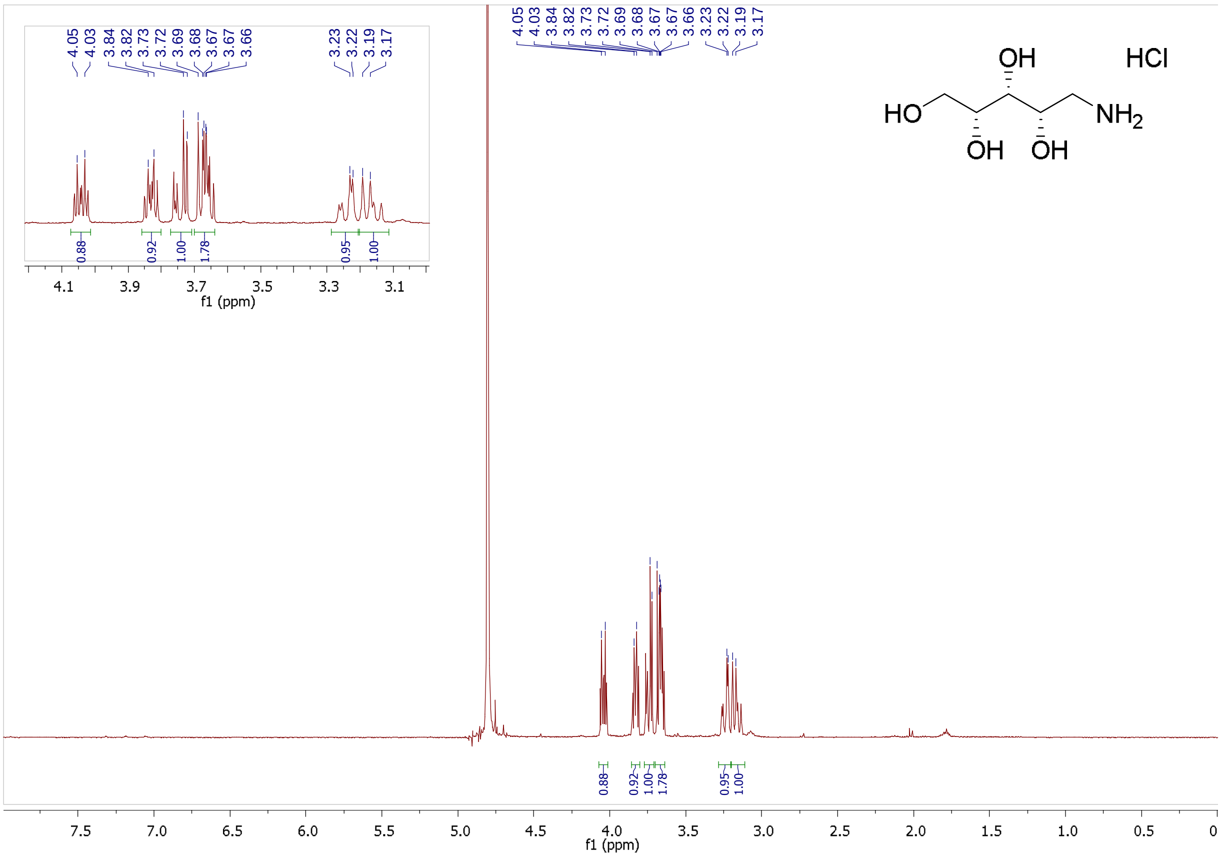

**2-Amino-2-deoxy-D-mannitol hydrochloride[14] (9)**

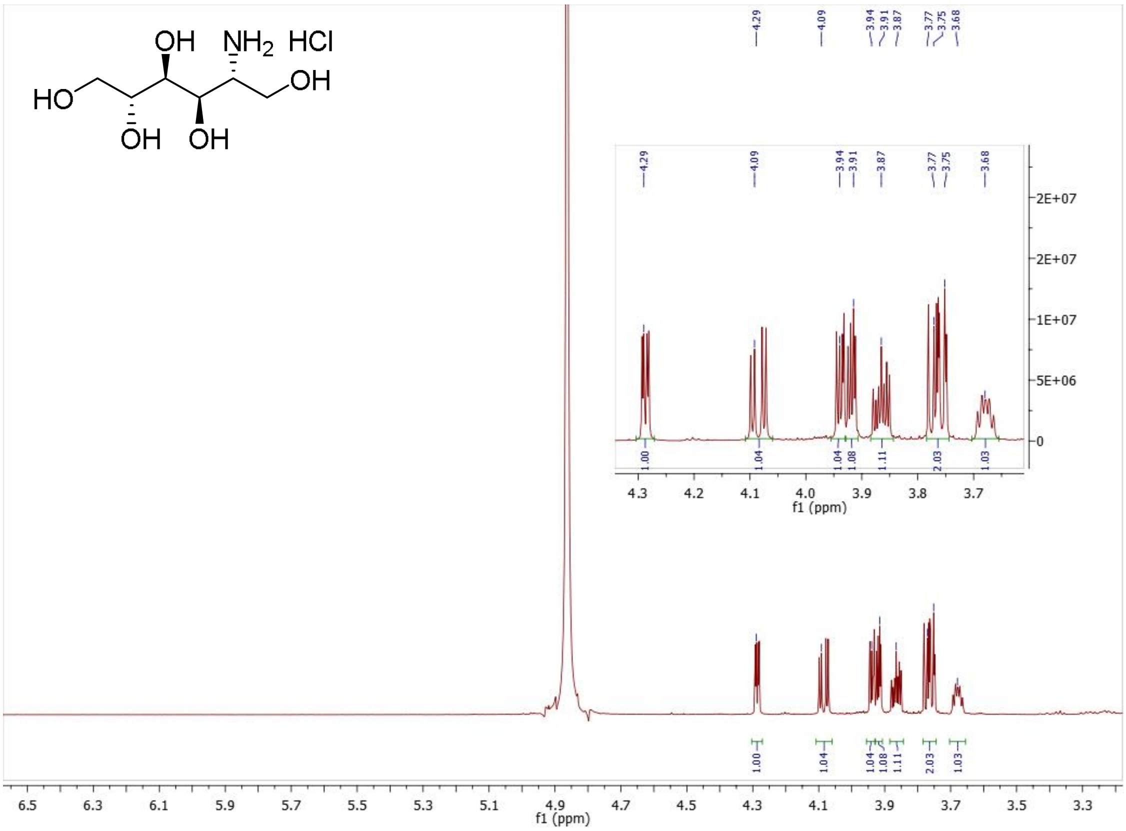


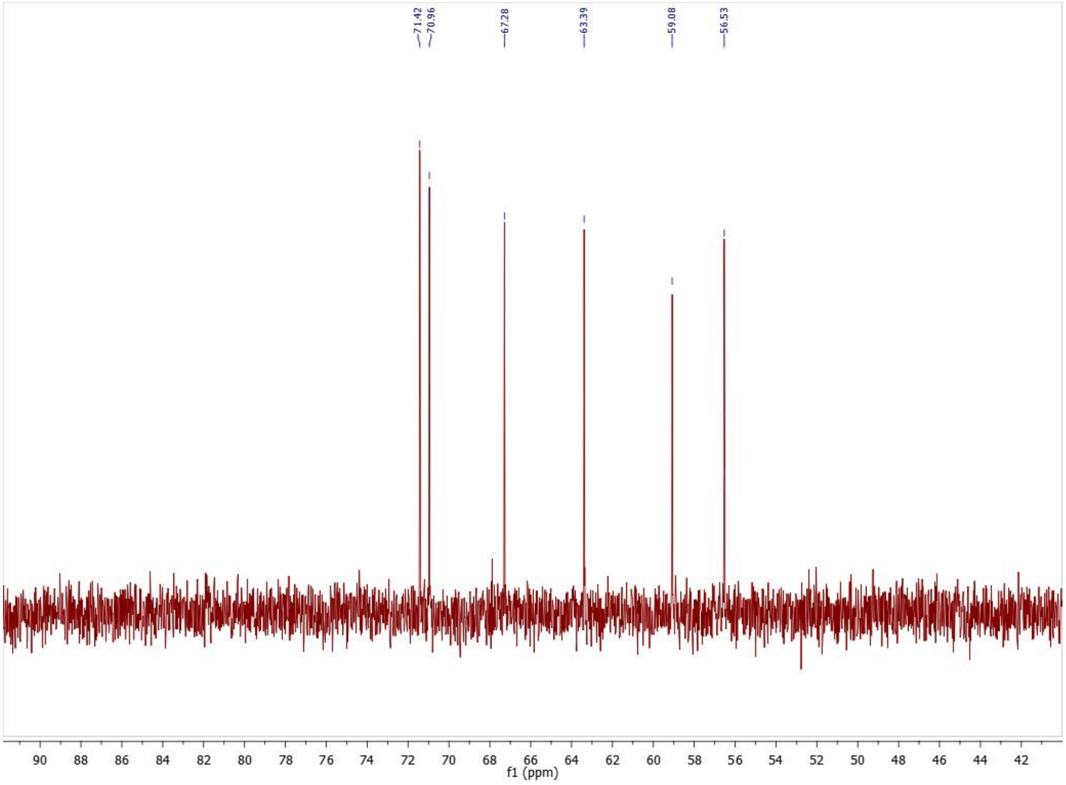

**2-Amino-2-deoxy-D-glucitol hydrochloride (*epi*-9)**

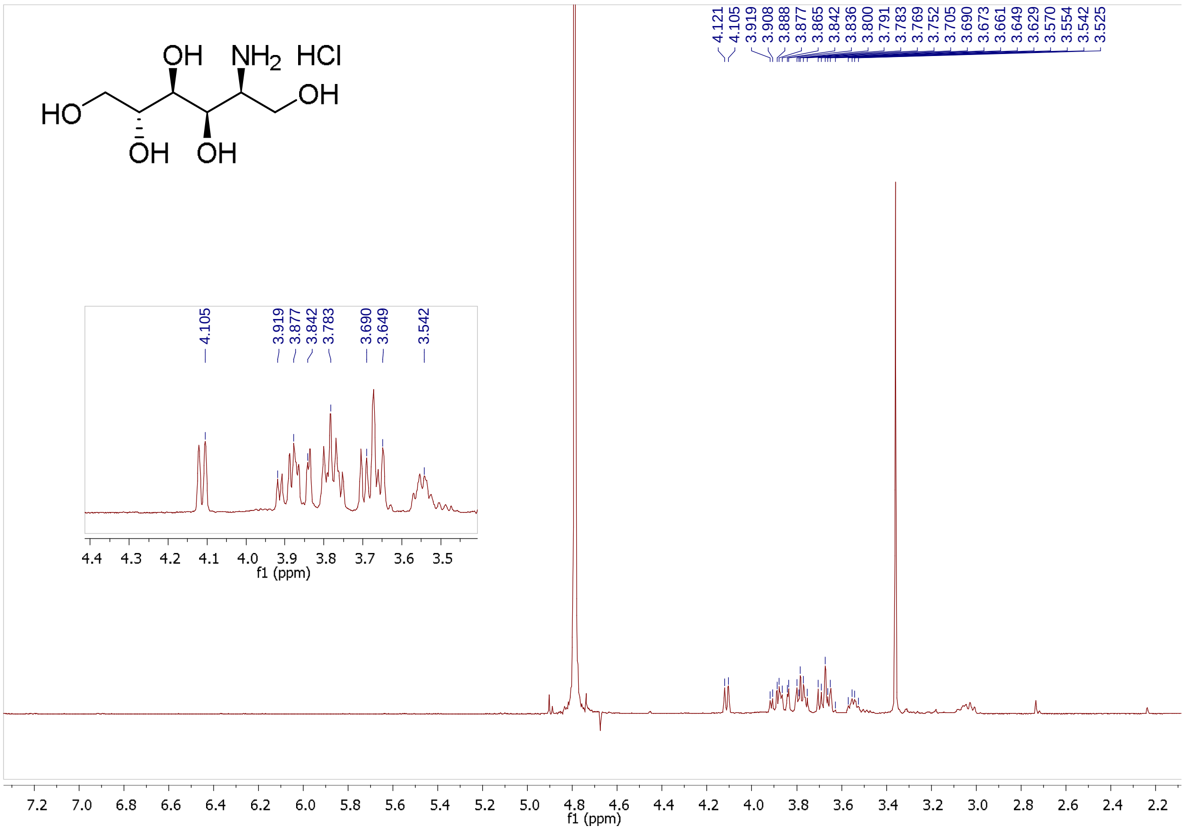


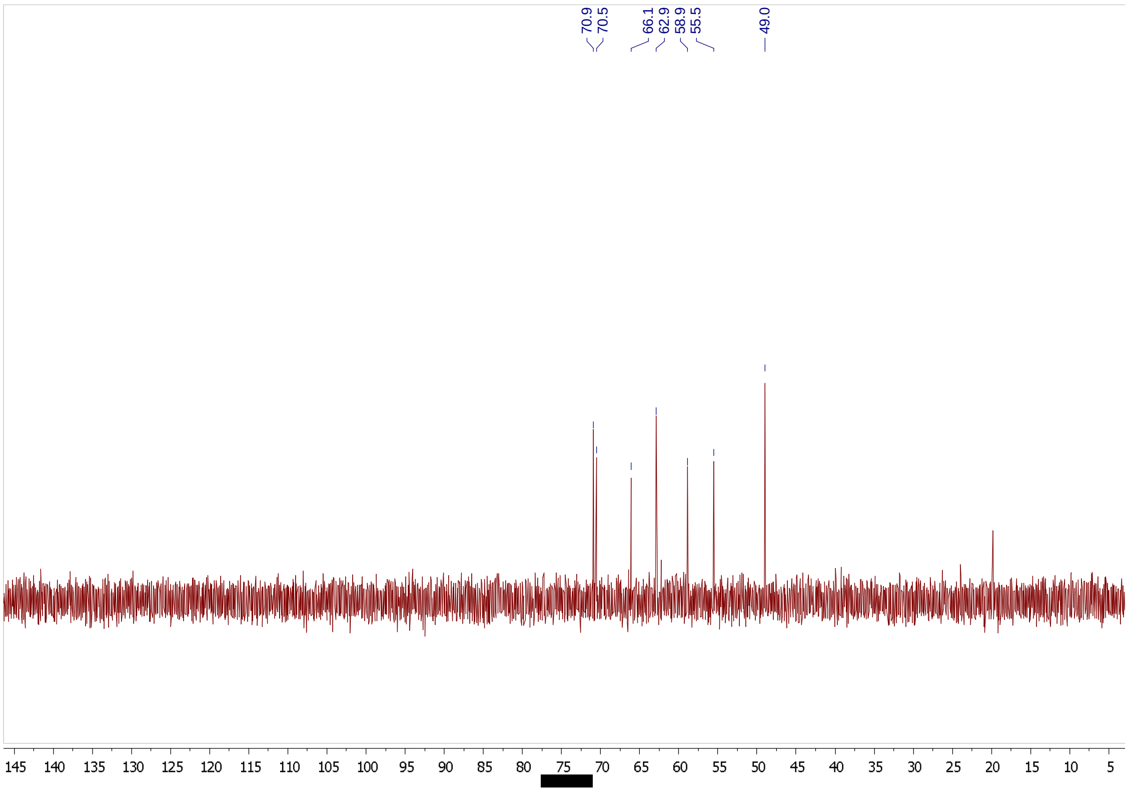

# VI. References


[1] L. Leipold, D. Dobrjevic, J. W. E. Jeffries, M. Bawn, T. S. Moody, J. M. Ward, H. C. Hailes, *Green Chem*. **2019***, 21*, 75-80.

[2] U. Kaulmann, K. Smithies, M. E. B. Smith, H. C. Hailes, J. M. Ward, *Enzyme Microb. Technol.* **2007**, *41*, 628ï 637.

[3] C. U. Ingram, M. Bommer, M. E. B. Smith, P. A. Dalby, J. M. Ward, H. C. Hailes, G. J. Lye, *Biotechnol. Bioeng.* **2007**, *96*, 559ï 569.

[4] Provided by J. M. Ward.

[5] M. F. Villegas-Torres, R. J. Martinez-Torres, A. Cazeres-Korner, H. Hailes, F. Baganz, J. Ward, *Enzyme Microb. Technol.* **2015**, *81*, 23ï 30.

[6] M. Höhne, S. Schätzle, H. Jochens, K. Robins, U. T. Bornscheuer, *Nat. Chem. Biol.* **2010**, *6*, 807ï 813.

[7] C. K. Savile, J. M. Janey, E. C. Mundorff, J. C. Moore, S. Tam, W. R. Jarvis, J. C. Colbeck, A. Krebber, F. J. Fleitz, J. Brands, P. N. Devine, G. W. Huisman, G. J. Hughes, *Science* **2010**, *329*, 305ï 310.

[8] pQR959 and pQR1017 were provided by R. J. Martinez-Torres (R. J. Martinez-Torres, J. M. Ward, *et al unpublished*) .

[9] J. S. Shin, H. Yun, J. W. Jang, I. Park, B. G. Kim, Appl. Microbiol. Biotechnol. 2003, 61 463ï 471.

[10] pQR805 was provided by U. Schnell (U. Schnell, J. M. Ward, *unpublished*).

[11] R. W. Foster, C. J. Tame, D. K. Buļ ar, H. C. Hailes, T. D. Sheppard, *Chem. - A Eur. J.* **2015**, *21*, 15947ï 15950.

[12] J. C. Norrild, C. Pedersen, J. Defaye, *Carbohydr. Res.* **1996**, *291*, 85ï 98.

[13] E. M. Dangerfield, C. H. Plunkett, A. L. Win-Mason, B. L. Stocker, M. S. M. Timmer, *J. Org. Chem*. **2010**, *75*, 5470ï 5477.

[14] Y. Wu, J. Arciola, N. Horenstein, *Protein Pept. Lett.* **2014**, *21*, 10ï 14.
